# ALG3 contributes to stemness and radioresistance through regulating glycosylation of TGF-β receptor II in breast cancer

**DOI:** 10.1186/s13046-021-01932-8

**Published:** 2021-04-30

**Authors:** Xiaoqing Sun, Zhenyu He, Ling Guo, Caiqin Wang, Chuyong Lin, Liping Ye, Xiaoqing Wang, Yue Li, Meisongzhu Yang, Sailan Liu, Xin Hua, Wen Wen, Chao Lin, Zhiqing Long, Wenwen Zhang, Han Li, Yunting Jian, Ziyuan Zhu, Xianqiu Wu, Huanxin Lin

**Affiliations:** 1 Department of Medical Oncology, Sun Yat-sen University Cancer Center, Guangzhou, 510060 Guangdong People’s Republic of China; 2 Department of Radiotherapy, Sun Yat-sen University Cancer Center, Guangzhou, 510060 Guangdong People’s Republic of China; 3 Department of Nasopharyngeal Carcinoma, Sun Yat-sen University Cancer Center, Guangzhou, 510060 Guangdong People’s Republic of China; 4 Department of Medical Oncology, The Sixth Affiliated Hospital of Sun Yat-Sen University, Guangzhou, 510655 Guangdong People’s Republic of China; 5 Department of Experimental Research, State Key Laboratory of Oncology in Southern China, Sun Yat-sen University Cancer Center, Guangzhou, 510060 Guangdong China; 6 Department of Experimental Research, The Seventh Affiliated Hospital of Sun Yat-sen University, Shenzhen, 518107 Guangdong People’s Republic of China; 7 Department of Radiotherapy, Nanfang Hospital, Guangzhou, 510515 Guangdong People’s Republic of China; 8 Department of Physiology, Sun Yat-sen University, Guangzhou, 510080 Guangdong People’s Republic of China; 9 Department of Gynecological Oncology, Sun Yat-sen University Cancer Center, Guangzhou, 510060 Guangdong People’s Republic of China; 10 Department of General surgery, The Third Affiliated Hospital of Guangzhou Medical College, Guangzhou, 510150 Guangdong People’s Republic of China; 11 Clinical Experimental Center, Jiangmen Key Laboratory of Clinical Biobanks and Translational Research, Jiangmen Central Hospital, Affiliated Jiangmen Hospital of Sun Yat-sen University, Jiangmen, 529030 Guangdong People’s Republic of China

**Keywords:** Breast cancer, ALG3, Radioresistance, Stemness, Glycosylation

## Abstract

**Background:**

Radiotherapy is a conventional and effective local treatment for breast cancer. However, residual or recurrent tumors appears frequently because of radioresistance. Novel predictive marker and the potential therapeutic targets of breast cancer radioresistance needs to be investigated.

**Methods:**

In this study, we screened all 10 asparagine-linked glycosylation (ALG) members in breast cancer patients’ samples by RT-PCR. Cell viability after irradiation (IR) was determined by CCK-8 assay and flow cytometry. The radiosensitivity of cell lines with different ALG3 expression was determined with the colony formation assay by fitting the multi-target single hit model to the surviving fractions. Cancer stem-like traits were assessed by RT-PCR, Western blot, and flow cytometry. The mechanisms of ALG3 influencing radiosensitivity was detected by Western blot and immunoprecipitation. And the effect of ALG3 on tumor growth after IR was verified in an orthotopic xenograft tumor models. The association of ALG3 with prognosis of breast cancer patients was confirmed by immunohistochemistry.

**Results:**

ALG3 was the most significantly overexpressing gene among ALG family in radioresistant breast cancer tissue. Overexpression of ALG3 predicted poor clinicopathological characteristics and overall survival (OS), and early local recurrence-free survival (LRFS) in breast cancer patients. Upregulating ALG3 enhanced radioresistance and cancer stemness in vitro and in vivo. Conversely, silencing ALG3 increased the radiosensitivity and repressed cancer stemness in vitro, and more importantly inhibition of ALG3 effectively increased the radiosensitivity of breast cancer cells in vivo. Mechanistically, our results further revealed ALG3 promoted radioresistance and cancer stemness by inducing glycosylation of TGF-β receptor II (TGFBR2). Importantly, both attenuation of glycosylation using tunicamycin and inhibition of TGFBR2 using LY2109761 differentially abrogated the stimulatory effect of ALG3 overexpression on cancer stemness and radioresistance. Finally, our findings showed that radiation played an important role in preventing early recurrence in breast cancer patients with low ALG3 levels, but it had limited efficacy in ALG3-overexpressing breast cancer patients.

**Conclusion:**

Our results suggest that ALG3 may serve as a potential radiosensitive marker, and an effective target to decrease radioresistance by regulating glycosylation of TGFBR2 in breast cancer. For patients with low ALG3 levels, radiation remains an effective mainstay therapy to prevent early recurrence in breast cancer.

**Supplementary Information:**

The online version contains supplementary material available at 10.1186/s13046-021-01932-8.

## Background

Breast cancer is the most common type of cancer diagnosed in females, and is the second leading cause of cancer-related death among women worldwide [1]. Radiotherapy (RT) remains a mainstay therapy for breast cancer after surgery, not only improving local control of cancer growth, but also reducing the risk of distant metastasis [2]. However, a significant proportion of breast cancer patients (about 33.8% percent in 1–3 node positive breast cancer patients) relapse within 10 years after radiotherapy [3], leading to a poor overall survival (OS) [4] due to radioresistance. Therefore, it is of paramount importance to explore the underlying mechanism responsible for radioresistance, which will facilitate to improve survival time in breast cancer patients.

Cancer stem cells (CSCs) are considered to contribute to radioresistance of tumors [5] due to several genetic and cellular adaptations, such as efficient DNA repair, hypoxia microenvironment and cell cycle blocked in the G0/G1-phase [6]. The expression of core pluripotency stem cell genes, including Homeobox Transcription Factor Nanog (NANOG), octamer-binding transcription factor 4 (OCT4) and sex determining region Y-box 2 (SOX2), play an important role in regulating self-renewal and multi-lineage differentiation of cancer stem cells. Thus, NANOG, OCT4 and SOX2 are often used as indicators for stem-like phenotype [7]. The expression of Ki67, cyclin B1, cyclin B2 [8] and CDK4 [9] influence the cell proliferation and the cell cycle, respectively. Within the stem cell compartment, these markers detect the subpopulation that is proliferating. Regulation of cancer stemness is complex, influenced by many factors. Multiple studies have reported that the transforming growth factor-β (TGF-β) pathway is associated with maintaining stem cell properties of breast cancer CSCs (BCSCs) [10–12]. TGF-β receptors (TGFBRs) play a critical role in TGF-β pathway activation. Post-translational modification of TGFBRs, including glycosylation [13], have attracted increasing attention, which has been reported to play a critical role in the protein function.

Glycosylation has a huge impact on every cellular process, such as recognition, signaling, cell matrix interaction and so forth [14]. Minor alterations in glycan structures significantly impact the biology of a cell, as well as treatment efficacy [15, 16], and targeting glycosylation has been considered a new road for cancer drug discovery [17]. Glycosylation is mediated by the coordinated action of several different glycosyltransferase and glycosidase enzymes [18]. Differential expression of glycosyltransferases within the tumor cells have been studied as predictive markers and potential therapeutic targets [19–21].

Glycans covalently attached to a polypeptide backbone of glycosylated proteins via nitrogen or oxygen linkages, known as N-glycans or O-glycans, respectively. And N-linked glycans branching increased during malignant transformation and is the most relevant cancer-associated carbohydrate structures [18, 22]. N-linked glycans play an important role in a spectrum of key biological processes in malignancy, including cell survival, growth, migration, metastasis, and host antitumor immunity [23, 24]. Glycoconjugates, such as glycosylated proteins and glycolipids, are generally located on cellular surface, and aberrant glycosylation often leads to the dysfunction of recognizing the extracellular factors and signal transduction [25]. TGFBRs I and II are transmembrane proteins, of which N-linked glycosylation is critical for membrane localization and function [26]. However, the mechanisms of TGFBRs glycosylation alteration remains unclear in breast cancer.

The core of N-linked glycans (N-glycans), Glc3Man9GlcNAc2, is comprised of two N-acetylglucosamine, nine mannose and three glucose residues [27]. The N-acetylglucosamine (GlcNAc) residue links to asparagine and additional sugar residues in the glycan depending on whether the glycosylation is high-mannose hybrid or complex type which is mediated by a series of glycosyltransferases, called “asparagine-linked glycosylation” (ALG) on a dolichol-pyrophosphate carrier [28]. Before being properly folded, the proteins carrying Glc1Man9GlcNAc2 are recognized by the endoplasmic reticulum (ER) chaperones, calnexin and calreticulin [29] where Asn-linked glycosylation 3 (ALG3; α-1,3-mannosyltransferase) catalyzes the first mannosylation to the lipid-linked Man5GlcNAc2.

ALG3 (found at 3q27.1, 30] is associated with early N-glycans synthesis [28] and located in the endoplasmic reticulum and the Golgi apparatus. ALG3 contributes to high-mannose type N-glycans in tumor cells. Aberrant expression of several high-mannose type N-glycans has been described associated with cancer progression [30]. The abundant high mannose glycans are detected in patients’ sera of breast cancer [31]. ALG3 is reportedly overexpressed in esophageal squamous cell carcinoma and squamous cell cervical carcinoma [32, 33] and causes drug resistance in myeloid leukemia [34]. ALG3 mutation results in under-glycosylation of glycosylated proteins and glycoprotein dysfunction [35, 36]. Studies have demonstrated that N-linked glycosylation of TGFBRs affects ligand-binding sensitivity [37]. However, the clinical significance and functional role of ALG3 in radioresistance of breast cancer need to be further elucidated.

In the present study, our results demonstrated that ALG3 was remarkably upregulated in radioresistant breast cancer tissue, which predicted poor clinicopathological characteristics and OS, and poor local recurrence-free survival (LRFS) in breast cancer patients. Upregulating ALG3 enhanced, while silencing ALG3 attenuated, proliferation, stemness and radioresistance in vitro and in vivo. Mechanistic investigation showed that ALG3 promoted radioresistance and cancer stemness by inducing glycosylation of TGF-β receptor II (TGFBR2). Importantly, both glycosylation blocker, tunicamycin, and TGFBR2 inhibitor, LY2109761, reduced cancer stemness and radioresistance in ALG3-overexpressing breast cancer cells. Our findings further suggested that ALG3 may serve as an effective target in breast cancer patients with high ALG3 levels. For patients with low ALG3 levels, radiation remains an effective mainstay therapy to prevent early recurrence in breast cancer. Therefore, our findings provide convincing evidence that ALG3 serves as a promising target to enhance sensitivity of breast cancer to radiotherapy.

## Material and methods

### Cell lines

Breast cancer cell lines MCF-7, MDA-MB-453, T47D and MDA-MB-231 were cultured in Dulbecco’s modified Eagle medium (DMEM) with 10% fetal bovine serum (FBS). BT549, HCC1937, and ZR-75-30 cell lines were cultured in Roswell Park Memorial Institute 1640 with 10% FBS. The MDA-MB-361 cell line was cultured in Leibovitz’s L-15 medium with 10% FBS, and the SK-BR-3 cell line was cultured in McCoy’s 5A medium with 10% FBS. All cell lines were purchased from American Type Culture Collection (Manassas, VA, USA). The SUM159PT cell line was purchased from ProCell (Wuhan, China) and cultured in Ham’s F-12 with 5% FBS, 1 μg/ml of hydrocortisone, 5 μg/ml of insulin, 10 mM HEPES, and 2 mM L-Glutamine. All experiments were performed with mycoplasma-free cells.

### Vectors and retroviral infection

Plenti-CMV-puro-P2A-3Flag-spCas9 and Plenti-U6-spg RNA (ALG3)-CMV-EGFP-P2A-blasticidin lentiviruses were purchased from BIiO Technology (Shanghai, China). Endogenous ALG3 knockout was performed according to the manufacturer’s instructions. Briefly, cells were seeded in 24-well plates with a density of 5 × 10^4^ cells per well. When the cells reached ~ 30–40% confluence, they were cultured in basic DMEM containing spCas9 lentiviruses and 5 μg/ml of polybrene for 2 h. Subsequently, completed DMEM containing 10% FBS and 5 μg/ml of polybrene was added and cultured for 12 h. Then, the culture medium was changed with fresh completed DMEM containing 10% FBS. Three days later, puromycin (0.25 μg/ml for SUM159PT and 4 μg/ml for MDA-MB-231) was used to select cells for 5 days. Western blotting was used to confirm whether the selected cells overexpressed spCas9. The selected cells were infected again with lentiviruses containing spgRNA (ALG3), and 3 days later, blasticidin (10 μg/ml for SUM159PT and 15 μg/ml for MDA-MB-231) was used to select cells for 5 days. Western blot was used to identify whether spgRNA (ALG3) was overexpressed in selected cells. After selection, cells were serially diluted in 96-well plates. Human ALG3 complementary DNA (cDNA) were amplified with Real time polymerase chain reaction (RT-PCR) and cloned into the pMSCV-puro-retro vector (Clontech, Beijing, China). Retroviral production and infection were performed, as described previously [38]. Stable cell lines expressing ALG3 were selected for 10 days by treatment with 0.5 μg/ml puromycin for 48 h after infection. Western blot confirmed that stable cell lines were constructed successfully.

### Patients and tissue samples

In this study, we used 376 paraffin-embedded breast cancer tissue samples, clinically and pathologically diagnosed at the Sun Yat-Sen University Cancer Center from 2008 to 2012. The clinical and pathological classification and stage were determined according to the 8th edition of the American Joint Committee on Cancer (AJCC) [39]. Histological grade was determined according to the Elston–Ellis modification of the Scarff–Bloom–Richardson system [40]. All 30 patients used to detect expression of ALG family were diagnosed with T2N2M0 breast cancer, who underwent radical mastectomy followed by adjuvant medical therapy and adjuvant radiotherapy delivering 50 Gy in 25 fractions over 5 weeks. And no residual tumor was seen on imagining after the radical mastectomy. As shown in Supplementary Figure S1A, these 30 patients are classified into Luminal (estrogen-receptor and/or progesterone-receptor positive), HER2 (estrogen-receptor and progesterone-receptor negative, HER2 positive), and triple-negative (estrogen-receptor, progesterone-receptor and HER2 negative) subgroups. And p53 status was also summarized in the Supplementary Table S1. The primary evaluation indicator for radiosensitivity were OS and LRFS. All patients provided prior informed consent, and the study was approved by the Institutional Research Ethics Committee. All the tissues were obtained the first time they were diagnosed.

### X-ray treatment

Cells in-96 wells plates and mice bearing tumors received X-ray treatment using an RS2000 X-ray Biological Research Irradiator (3 mm copper filter, 160 kV, 25 mA, Rad Source Technologies, GA, USA) at Sun Yat-Sen University Cancer Center. Dosimetry was performed semiannually using an ionization chamber connected to an electrometer system that was directly traceable to a National Institute of Standards and Technology calibration. The mice were narcotized with 60 mg/kg of pentobarbital injected in the abdominal cavity. A 2-mm Cu filter was used for in vivo xenograft experiments.

### Real-time RT-PCR

Total RNA samples from the breast cancer cell lines and breast cancer tissues were extracted using TRIzol (Invitrogen, Carlsbad, CA, USA) according to the manufacturer’s instructions. The extracted RNA was pretreated with RNase-free DNase, and ~ 2 μg of RNA from each sample was used for cDNA synthesis primed with random hexamers. For RT-PCR amplification of ALG3 cDNA, an initial amplification step using ALG3-specific primers was performed with activation at 95 °C for 10 min, followed by 40 cycles of denaturation at 95 °C for 15 s, primer annealing/extension at 60 °C for 60 s. Next, a final step to melting curve analysis at 95 °C for 10 s, 65 °C for 5 s and 95 °C for 30 s was performed before the reaction mixture was stored at 4 °C. Real-time RT-PCR was used to determine the increase in ALG3 messenger RNA in the primary breast cancer tissue samples. The primers were designed using Primer Express v 2.0 software (Applied Biosystems, Foster City, CA, USA). The forward and reverse primer sequences are shown as Table 1. Expression data were normalized to the geometric mean of the housekeeping gene GAPDH to control variability in expression levels and were calculated as 2^[(GAPDHCq) − (ALG3Cq)], where Cq represents the threshold cycle value for each transcript.

**Table 1:** Gene primers

Gene Name	Forward primer	Reverse primer
ALG3	5′CACCGTTAAGATGGCGGCT3′	5′CCATTGCTTGCAGAGTCCCT3′
ALG1	5′CATGTAGTAGCGGTGGTGCT3′	5′GCCCAACTGCAAGACTCTGA3′
ALG2	5′GTACATGGCTCCCCATTGGT3′	5′TTGGGGCCCTGTATAGTCGT3′
ALG6	5′ATCTTGTGACTGCGACCTCC3′	5′GGCAAGGCGTGGTAAAGTTC3′
ALG8	5′CCCCACATACCATTCCACAGA3′	5′CGTCCACTCTGAAGTTGCCT3′
ALG9	5′CGACTTCATAGGGTGCCGAA3′	5′CCAGATAACTCGGTCCGGTG3′
ALG10	5′TTCCAGGAGTAGGTTCTTGGGC3′	5′GGCCGAGAAATAGTAACCTTCCA3′
ALG11	5′ GTGCCTGTGCAAGTTGTTGAG3′	5′GTAGCAGCAGTCTGATTCCCC3′
ALG12	5′ GGCCCTGTATGTGTCCCATT3′	5′GCACATCCTCCCTCTTGTCG3′
ALG13	5′TGTTACCGTAGGGACCACCA3′	5′TCAGGTACCACCGTTCCTCT3′
FOXD1	5′TTCTCGTCTTGGTGGTTCGG3′	5′CAAACGTCAAGGGAGCCTCT3′
SPHK1	5′GAGCGAAAAGTTTGAGGCCG3′	5′GTTCCCTACAGTGGCCTGG3′
CHST11	5′ATGCGGAGGAATCCCTTTGG3′	5′GGTCCTCATCCACCACCAAG3′
EST2	5′CAAGCTGTTTGCGGGGATTC3′	5′TCCGGGCATAGCTGAGGAAG3′
PIK3CD	5′CATTCCTCCTCCATCCTCGC3′	5′AGCTCCTCCTCGGTGACAT3′
TBX3	5′ACTTCCTGCACCAACACCAA3′	5′CCTCCCCACAGCAATCTCAG3′
PPARD	5′GCACCAACGAGGGTCTGGAA3′	5′CGGATCGTACGACGGAAGAA3′
BHLHE40	5′TGGAGCCTTCCTGAAGGTGTAA3′	5′GGACATGGGAGTCAGCCATA3′
GAPDH	5′TTGAGGTCAATGAAGGGGTC3′	5′GAAGGTGAAGGTCGGAGTCA3′

### Western blotting

Western blotting was performed, as described previously [41], using antibodies against ALG3 (NO. 20290–1-AP, PROTEINTEC, Manchester, UK), TGFBR2(NO. 79424, Cell Signaling Technology, Inc., Beverly, Massachusetts, USA), TGFBR1(NO. ab31031, Abcam, Inc., Cambridge science park, UK), phosphorylated SMAD2 (NO. 55041, Cell Signaling Technology, Inc., Beverly, Massachusetts, USA), SMAD2 (NO. 5339, Cell Signaling Technology, Inc., Beverly, Massachusetts, USA), NANOG (NO. 3580, Cell Signaling Technology, Inc., Beverly, Massachusetts, USA), SOX2 (NO. 4900, Cell Signaling Technology, Inc., Beverly, Massachusetts, USA), OCT4 (NO.2750, Cell Signaling Technology, Inc., Beverly, Massachusetts, USA), CyclinB1 (NO. 4135, Cell Signaling Technology, Inc., Beverly, Massachusetts, USA), CyclinB2 (NO. 21644–1-AP, Proteintech Group, Inc., Rosemont, IL, USA) and CDK4 (NO. 12790, Cell Signaling Technology, Inc., Beverly, Massachusetts, USA). Anti-α-Tubulin mouse monoclonal antibody (NO. 66031–1-Ig, Proteintech Group, Inc., Rosemont, IL, USA) was used to confirm equal loading.

### Immunofluorescence staining

Cells were washed by PBS and then fixed with 4% paraformaldehyde for 15 min at 37 °C. After fixation cells were rinsed with PBS, the cells were blocked with 0.1% Triton X-100 containing 1% bovine serum albumin in PBS for 1 h. This was followed by incubation in antibody against TGFBR2 (NO. AF0259, Affinity Biosciences, Cincinnati, USA) and p-smad2 (AF8314, Affinity Biosciences, Cincinnati, USA) for 16 h at 4 °C in a humidified chamber. After washed with PBS, cells were incubated for 1 h at room temperature with fluorescently labeled secondary antibodies. Finally, cells were rinsed in PBS, covered slip with DAPI and examined with a confocal microscope (C1 si, Nikon, Japan).

### Caspase-9 or Caspase-3 activity assays

We used the Caspase-9 Colorimetric Assay Kit or Caspase-3 Colorimetric Assay Kit (Keygen, China) to analyze activity of caspase-9 or caspase-3 by spectrophotometry. Firstly, breast cancer cells were suspended in 1 ml ice-cold PBS and washed twice, and then resuspended in lysis buffer. After 30 min incubation on ice, we mixed 50 μl cell suspension, 50 μl reaction buffer, and 5 μl caspase-3/9 substrate, and then incubated it at 37 °C for 1.5 h. The absorbance was measured at 405 nm.

### Immunoprecipitation

Cell lyses for protein extraction and co-immunoprecipitation were performed as follows. Cells were lysed in RIPA buffer (150 mM NaCl, 5 mM MgCl_2_, 20 mM Tris-HCl [pH 8.0], 1%Triton X-100 and 0.5% deoxycholate) with complete mini protease inhibitor (Roche). For immunoprecipitation of protein complexes, cell extracts were pre-cleared with Protein G agarose beads (Millipore, 16–266). Then, incubated cell extracts with the polyclone rabbit antibody to TGFBR1 (NO. sc518045, Santa Cruz Biotechnology, Inc., California, USA) or TGFBR2 (NO. ab225902, 1:100, Abcam, Inc., Cambridge science park, UK) for 16 h at 4 °C. Add beads in the cell extracts and incubated for 3 h at 4 °C. Rinsing beads with equilibrium buffer and the protein extraction were then dealt with as for western blot.

### Luciferase assay

Cells were seeded in 24-well plates at a density of 2 × 10^4^ cells per well for 24 h. Then, they were transfected with 100 ng of luciferase reporter plasmids or the control-luciferase plasmid, plus 5 ng of pRL-TK *Renilla* plasmid (Promega, Madison, USA), using the Lipofectamine 3000 reagent (Invitrogen), according to the manufacturer’s instructions. After 24 h, firefly and Renilla signals were measured using the Dual Luciferase Reporter Assay Kit (Promega, Madison, Wisconsin, USA). ARE-luciferase reporter plasmid (Genomeditech, Shanghai, China), HRE-luc (Genomeditech, Shanghai, China), NF-κB-luc (Genomeditech, Shanghai, China), FOXO-luc (Genomeditech, Shanghai, China), SMAD-luc (Genomeditech, Shanghai, China) and TCF-1-luc (Genomeditech, Shanghai, China) were selected to examine activation of antioxidant, hypoxia, NF-κB, PI3K/AKT, TGF-β signaling, and Wnt pathways.

### Immunohistochemistry (IHC)

IHC staining and analysis of ALG3 using antibodies against ALG3 (NO. ab151211, Abcam, Inc., Cambridge science park, UK) were performed, as described previously [39, 42]. The ALG3 staining index was analyzed on the basis of the staining intensity (1: no or weak staining; 2: moderate staining; 3: strong staining) and extent (0, 0–25%; 1: 25–50%; 2: 50–75%; 3: 75–100%). A staining score was calculated by multiplying the staining intensity score and the positive cell percentage. The staining intensity and extent values were multiplied and scored as 0, 1, 2, 3, 4, 6, 8 or 9. Using this scoring method, we assessed ALG3 expression in the 376 breast cancer tissue samples. The mean optical density method was used to count inconsistent IHC staining intensities. The best cutoff value was determined using the log-rank test with respect to OS.

### Cell counting Kit-8 (CCK-8) assay

Cell viability after radiation was evaluated with Cell Counting Kit-8 (CCK-8; Beyotime, China). MCF-7, ZR-75-30, SUM159PT, and MDA-MB-231 cells were seeded in 96-well plates for 24 h. Then the plates were exposed to 0, 2, 4, 6, (8, 10) Gy X ray. After 72 h, 10 μl of CCK-8 solution was added to each well and incubated for 2 h. The absorbance was measured at a wavelength of 450 nm using a microplate reader (Bio-Rad Laboratories, California, USA). Cell viability was calculated using the following formula: cell viability = (optical density (OD) value of the treatment group/OD value of the control group) × 100%.

### Colony formation assay

Cells were seeded in 6-well plates with at a different density of cells per well and treated with 0, 2, 4, 6 Gy radiation. After 14 days, the cells were then fixed with methanol and the colonies stained with 0.4% crystal violet.

$$ \mathrm{Surviving}\ \mathrm{Fraction}=\mathrm{Colonies}/\left(\mathrm{Input}\ \mathrm{cells}\times \mathrm{Plating}\ \mathrm{Efficiency}\right) $$

$$ \mathrm{Plating}\ \mathrm{Efficiency}=\mathrm{Colonies}\ \mathrm{in}\ \mathrm{control}\ \mathrm{group}/\mathrm{Input}\ \mathrm{cells}\ \mathrm{in}\ \mathrm{control}\ \mathrm{group}\times 100\% $$

The mean surviving fraction and its standard error were calculated from 3 independent experiment for each cell line. Survival curves were fitted using GraphPad Program by nonlinear regression analysis based on multi-target single-hit model. Clone survival fraction (S) with increasing dose (D) can be described using S = 1 – (1 – e^−D/D0^) ^*N*^ = 1-(1-e^-kD^)^N^. Based on the results of the nonlinear regression analysis, we got k and N values. D_0_ = 1/k, and D_q_ = D_0_ × ln (N). N value represents the comprehensive degree of sub-damage repair capacity and the sensitivity to radiation damage. D_0_ value represents the sensitivity to radiation damage. The smaller the D_0_ value, the smaller the dose is required to kill a certain proportion of cells; Dq reflects the ability against DNA damage. The greater the Dq value, the greater the dose is required to induce cells death.

### Tumor sphere formation assays

Five hundred cells were seeded in 6-well ultra-low attachment plates and cultured in suspension in serum-free DMEM: F12 medium (BioWhittaker) supplemented with 0.4% BSA, 2% B27 (Invitrogen), 20 ng/ml of epidermal growth factor, 20 ng/ml of basic fibroblast growth factor, and 5 μg/ml of insulin (PeproTech, Rocky Hill, NJ, USA) for 10 days. The number of spheres formed (tight, spherical, nonadherent masses > 50 μm in diameter) was counted, and images were captured under an inverted microscope (C1 si, Nikon, Japan).

$$ \mathrm{Sphere}\ \mathrm{formation}\ \mathrm{efficiency}\ \left(\%\right)=\left(\mathrm{Colonies}/\mathrm{Input}\ \mathrm{cells}\right)\times 100 $$

### Flow cytometry assay

Cells were dissociated with trypsin, resuspended at 1 × 10^6^ cells/ml in DMEM containing 2% FBS, and then incubated at 37 °C for 30 min with or without 100 μM verapamil (Sigma-Aldrich, St. Louis, MO, USA) to inhibit adenosine triphosphate-binding cassette transporters. The cells were subsequently incubated with 5 μg/ml Hoechst 33342 (Sigma-Aldrich) at 37 °C for 90 min or stained at 4 °C with purified CD44 (ab157107, rabbit immunoglobulin G [IgG]; Abcam)–fluorescein isothiocyanate (FITC) (ab6717, rabbit IgG; Abcam), CD24 (ab31622, mouse IgG1; Abcam)-PE-CY7 (ab130790, mouse IgG2b; Abcam), or annexin V-FITC–propidium iodide (PI) (KGA106). Finally, the cells were incubated on ice for 10 min and washed with ice-cold phosphate-buffered saline prior to flow cytometry analysis. Samples were analyzed and sorted on Beckman–Coulter MoFlo and Beckman–Coulter gallios, respectively, with data analyzed using FlowJo software (Tree Star Inc., USA).

### Xenograft tumor model

A xenograft tumor model was constructed, as described previously [43]. Female BALB/c nude mice (4–5 weeks old, 18–20 g in weight) were purchased from Slac-Jingda Laboratory Animal (Hunan, China). All experimental procedures were approved by the Institutional Animal Care and Use Committee of Sun Yat-Sen University. The mice’s flanks were injected subcutaneously with 5 × 10^6^ MCF-7, ZR75–30, MDA-MB-231 and SUM159PT cells. The tumors were examined every week, including length and width, using calipers, and tumor volumes were calculated. After 14 days, the mice from ALG3-transduced, ALG3 vector, ALG3 knocked-out, and control groups underwent six fractions with 2 Gy/fraction radiation. Next, we compared the speed of tumor growth between groups with different ALG3 expression. On day 63, the mice were euthanized and the tumors excised and measured.

### Statistical analysis

Statistical analyses were performed using SPSS v22 statistical software (SPSS Inc., Chicago, IL, USA). Comparison between categorical variables was performed by chi-square test. Pearson or Spearman correlation test was used to examine correlations between two continuous variables when indicated. Survival curves were plotted using the Kaplan–Meier method and the log-rank test, and survival data were evaluated using multivariate cox regression analysis. *P* < 0.05 was considered statistically significant. Two-way ANOVA with Bonferroni’s post test, one-way ANOVA with Tukey’s multiple comparison test, and Student’s *t* test were used to evaluate the difference between the experimental group and the control group.

## Results

### ALG3 is significantly overexpressed in radioresistant breast cancer tissues

**Fig. 1:**
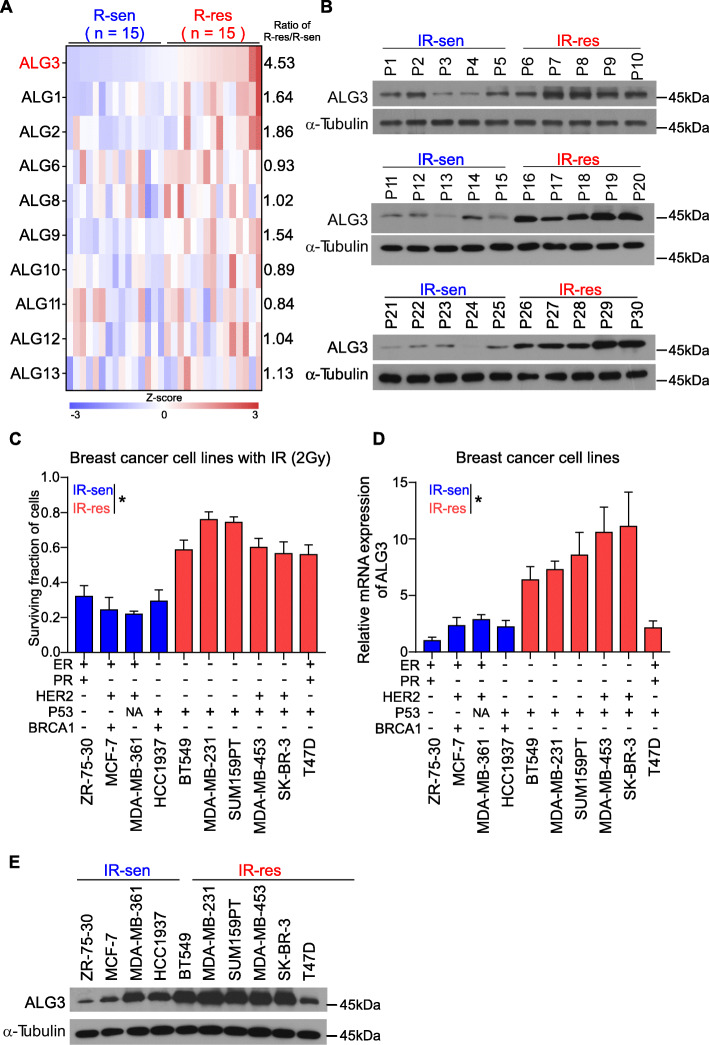
ALG3 was highly expressed in cancerous tissue and radioresistant breast cancer. **a** mRNA expression level of ALG family were detected by RT-PCR. GAPDH was detected as a loading control in PCR. **b** Protein expression of ALG3 in radioresistant and radiosensitive breast cancer tissue. α-Tubulin was detected as a loading control in Western blot. **c** Cell viability after radiation treatment of each cell line was examined by CCK-8 assays. Data were analyzed by Student’s t-test. Each bar represents the mean ± SD of three independent experiments. **d, e** RT-PCR and Western blot analysis of ALG3 expression in breast cancer cell lines. GAPDH was used as endogenous control in RT-PCR, and α-Tubulin was detected as a loading control in Western blot. Data were analyzed by Student’s t-test. Each bar represents the mean ± SD of three independent experiments. *P < 0.05

To explore a critical glycosyltransferase in radioresistance of breast cancer, we first analyzed the expression levels of all 10 members of the ALG family in 30 breast cancer tissues, including 15 radioresistance and 15 radiosensitivity breast cancer tissues stratified by the status of recurrence 5 years after initial radiotherapy: radioresistant (recurrence) or radiosensitive (no recurrence). As shown in Fig. 1a, expression levels of ALG1, ALG2, ALG3, ALG8, ALG9, ALG12 and ALG13 were differentially upregulated in radioresistant breast cancer tissues compared with those in radiosensitive tissues, particularly ALG3 with the highest level (4.53-fold change). ALG3 expression was further analyzed in 15 radioresistant patients and 15 radiosensitive breast cancer tissues using Western blot, and the results showed ALG3 expression was dramatically increased in the radioresistant group compared to that in the radiosensitive group (Fig. 1b). The detailed information of 30 patients were shown in Supplementary Table S1. And the distribution of different tumor type or TP53 status were shown in Supplementary Figure S1A (left). Moreover, we compared the ALG3 relative mRNA expression in TP53 mutation and TP53 wild-type subgroup, as shown in Supplementary Figure S1A (right). But no significant difference of ALG3 expression between TP53 mutation and TP53 wild-type subgroup was seen. Then we performed Chi-square test to analyze the association between radiosensitivity and the tumor type and the p53 status among 30 patients, the results suggested that the tumor type (*p* = 0.043) and the p53 status (*p* = 0.025) were significantly associated with the radiosensitivity (Supplementary Table S2). To further support our findings, we analyzed the association of ALG3 expression with tumor type, TP53 mutation, BRCA1 mutation and BRCA2 mutation in TCGA database. The results showed that the expression of ALG3 was markedly increased in the basal-like and human epidermal growth factor receptor 2(Her2) subtypes (Supplementary Figure S1B) compared with that in luminal subtype, both of which has been reported to show higher local recurrence rates after radiotherapy [44, 45]; and the expression of ALG3 was significantly increased in cases with TP53 mutation. No differences were found between BRCA carriers and BRCA-negative patients (Supplementary Figure S1C). The response of 10 breast cancer cell lines to irradiation (IR) was further investigated through CCK-8 assay. As shown in Fig. 1c, BT549, MDA-MB-231, SUM159PT, MDA-MB-453, SKBR3 and T47D were relatively radioresistant (surviving fraction > 0.5 after 2 Gy radiation), whereas ZR-75-30, MCF-7, MDA-MB-361 and HCC1937 were relatively radiosensitive (surviving fraction ≤0.5 after 2 Gy radiation), consistent with the findings in the previous study [46], where BT549, MDA-MB-231 are radioresistant cell lines, T47D is moderately resistant cell lines, while MCF-7 and ZR-75-30 are radiosensitive cell lines. Importantly, average expression levels of ALG3 in radioresistant breast cancer cell lines were significantly higher than those in radiosensitive breast cancer cell lines (Fig. 1d and e). Given the ALG3 overexpression in basal-like cells, including SUM159PT and MDA-MB-231 shown in Fig. 1d and e, we inferred that the correlation between ALG3 and radioresistance is more commonly seen in highly aggressive basal-like and Her2 subtypes. To further provide a proof that the protein level detected by WB was consistent with RNA level detected by PCR, we performed Pearson correlation analysis (r = 0.9157) between the gray-scale and the mRNA expression, which indicated that the expression of ALG3 in the 10 cell lines obtained using these two methods are consistent (Supplementary Figure S1D). Moreover, we further analyzed the cause of ALG3 overexpression from a genetic perspective in a breast dataset from The Cancer Genome Atlas (TCGA), and found that mRNA expression levels of ALG3 in breast cancer tissues with gain or amplification were robustly upregulated compared with those with deletion or diploid (Supplementary Figure S1E). Collectively, our results suggest that amplification or gain-induced ALG3 overexpression may play a crucial role in radioresistance of breast cancer.

### ALG3 contributes to radioresistance in vitro and in vivo

**Fig. 2:**
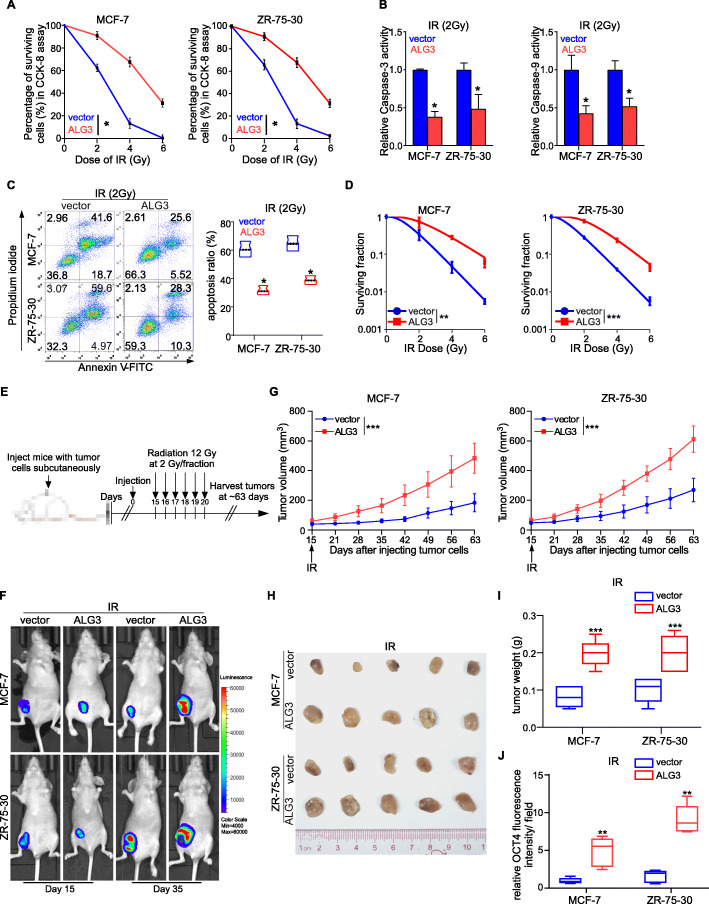
ALG3 overexpression contributes to radioresistance. **a** Surviving fraction of MCF- 7 and ZR-75-30 cells irradiated with 0, 2, 4, and 6 Gy X-rays and cultured for 72 h were detected by CCK-8 assays. Data were analyzed by two-way ANOVA with Bonferroni’s post test. Each bar represents the mean ± SD of three independent experiments. **b** Caspase-3 and Caspase-9 activity in MCF- 7 and ZR-75-30 cells. Data were analyzed by Student’s t-test. Each bar represents the mean ± SD of three independent experiments. **c** Apoptosis ratio of MCF-7 and ZR-75-30 cells after 2 Gy radiation treatment and cultured for 72 h was detected by flow cytometry. Data were analyzed by Student’s t-test. Each bar represents the mean ± SD of three independent experiments; *P < 0.05. **d** The dose–survival curve was fitted using a multitarget single-hit statistical model by colony assay. Data were analyzed by two-way ANOVA with Bonferroni’s post test. **e** Model of treatment schedule in vivo studies. **f** Representative fluorescence graph of tumor-bearing mice with different ALG3 expression, which were treated with 2 Gy X ray. **g** Tumor growth rate was significantly increased in ALG3 transduced cells after radiation treatment. Data were analyzed by two-way ANOVA with Bonferroni’s post test. **h** Representative pictures of tumors from vector + IR, and ALG3-trancedced + IR mice were as shown. **i** The tumor weight of the vector group after radiation treatment and the tumor weight of the ALG3-tranceduced group after radiation treatment were calculated and shown. Data were analyzed by Student’s t-test. **j** The fluorescence intensity of OCT4 in ALG3-transduced cells relative to that in vector cells were measured. Data were analyzed by Student’s t-test. *P < 0.05, **P < 0.01, *** *P* < 0.001

To investigate whether ALG3 affects radiosensitivity of breast cancer cells, we first established stably ALG3-overexpressing cell lines using MCF-7 and ZR-75-30 cells (Supplementary Figure S2A), both of which showed relative lower ALG3 expression and more sensitive to radiation treatment as indicated in Fig. 1c-1e. The effect of ALG3 on radiosensitivity was further determined by CCK-8 assay in vitro. First, breast cancer cells were exposed to 0, 2, 4, 6 Gy X-rays prior to submission to CCK-8. As shown in Fig. 2a, upregulating ALG3 could significantly increase survival rates in MCF-7 and ZR-75-30 cells in the presence of different dose of radiation. Furthermore, the activity of caspase-3 and caspase-9 was suppressed by ALG3-overexpression under 2 Gy radiation treatment (Fig. 2b). Conversely, the apoptotic ratio of ALG3-overexpressing cell was reduced compared to the vector control cells after radiation treatment (Fig. 2c). To further evaluate the effect of ALG3 on radioresistance, we performed colony formation assays for the different cell lines (Supplementary Figure S2B). The survival curves were fitted with the multitarget-single hit model (Fig. 2d). The mean surviving fraction and the *p* values between vector and ALG3-transduced group were shown in Supplementary Table S3–4. The mean radiobiological parameters were summarized in Table 2. The values of N, D_q_, D_0_, SF2 were higher in the ALG3-transduced group than that in the ALG3-vector groups, which indicated that significantly greater the dose was required to kill cells in ALG3-overexpressing group. These results indicate that ALG3 increases the surviving fraction and reduces apoptosis percentages of breast cancer cells, which suggest that ALG3 overexpression confers radioresistance to breast cancer cells.

**Table 2:** Radiobiological Parameters

Cell lines	N (Mean ± SD^a^)	D_0_ (Mean ± SD^a^)	D_q_ (Mean ± SD^a^)	SF2 (Mean ± SD^a^)
MCF-7-vector	3.06 ± 0.28	1.00 ± 0.05	1.12 ± 0.13	0.36 ± 0.05
MCF-7-ALG3	4.90 ± 0.82	1.42 ± 0.12	2.24 ± 0.05	0.74 ± 0.02
ZR-75-30-vector	2.04 ± 0.35	1.09 ± 0.09	0.76 ± 0.17	0.30 ± 0.04
ZR-75-30-ALG3	4.96 ± 1.22	1.40 ± 0.14	2.20 ± 0.14	0.73 ± 0.03
MDA-MB-231-control	5.48 ± 1.92	1.52 ± 0.14	2.50 ± 0.34	0.81 ± 0.08
MDA-MB-231-sg1	2.10 ± 0.17	1.20 ± 0.17	0.90 ± 0.15	0.36 ± 0.09
MDA-MB-231-sg2	1.63 ± 0.70	1.21 ± 0.08	0.50 ± 0.43	0.28 ± 0.07
SUM159PT-control	3.98 ± 0.44	1.52 ± 0.03	2.10 ± 0.13	0.71 ± 0.03
SUM159PT-sg1	1.59 ± 0.40	1.26 ± 0.06	0.55 ± 0.35	0.30 ± 0.06
SUM159PT-sg2	2.28 ± 0.09	1.17 ± 0.12	0.95 ± 0.15	0.36 ± 0.04

*N* extrapolation number, *D*_*0*_ mean lethal dose, *D*_*q*_ quasi-threshold Dose, *SF2* surviving fraction at 2 Gy

^a^Mean ± SD represents mean values of surviving fractions ± standard deviations

Next, orthotopic xenograft tumor model was used to investigate the effect of ALG3 overexpression on response of breast cancer cells to radiation in vivo. ALG3–transduced cells or vector cells were injected subcutaneously into the flanks of female mice. When the xenografts were palpable from day 14 after inoculation (approximately 0.5 cm in diameter), the mice received radiation treatment in doses of 2 Gy for 6 consecutive days (Fig. 2e). To protect the important soft-tissue organs of mice, we used lead sheets to cover the tissue other than the subcutaneous tumor (Supplementary Figure S2C). And fluorescence intensity images of mice injected with MCF-7 or ZR-75-30 cells are presented in Fig. 2f, which indicates that at day 35 after injection, the tumor growth rate was significantly increased in ALG3-transduced group under radiation treatment. To compare growth rate at different time point, the relative tumor volume was calculated at 1-week time interval, as shown in Fig. 2g. We observed that upregulation of ALG3 significantly promoted tumor growth rate of MCF-7 and ZR-75-30 compared with the vector group. All mice were sacrificed at day 63, and subcutaneous tumors were extracted (Fig. 2h). As shown in Fig. 2g and i, we found that ALG3 significantly increased tumor volume and weight compared with the vector group after radiation treatment. Moreover, OCT4 intensity was also upregulated in ALG3-overexpressing tumor tissues (Fig. 2j). The immunohistochemical (IHC) staining showed that ALG3 expression was dramatically higher in radioresistant compared with radiosensitive tumor tissue at the end of the experiment (Supplementary Figure S2D). Based on the results obtained in vitro (Fig. 2a-2d), we assumed that both a reduction in cell death and an increase in proliferation after radiation therapy contributed to the suppression of the subcutaneous tumor size. To further validate this speculation, immunohistochemical staining of Ki67 and cleaved caspase-3 (an activated pro-apoptotic protein of caspase family), was performed using the tumor tissues from mice. As shown in Supplementary Figure S2E-F, radioresistant tumor tissues have a higher level of Ki67 expression and showed a significant lower caspase-3 activity, which indicated that both the apoptosis and proliferation process were involved in ALG3 induced radioresistance. These results demonstrated that ALG3 promotes the resistance of breast cancer cells to radiation in vitro and in vivo.

### Downregulation of ALG3 radiosensitizes breast cancer cells

**Fig. 3:**
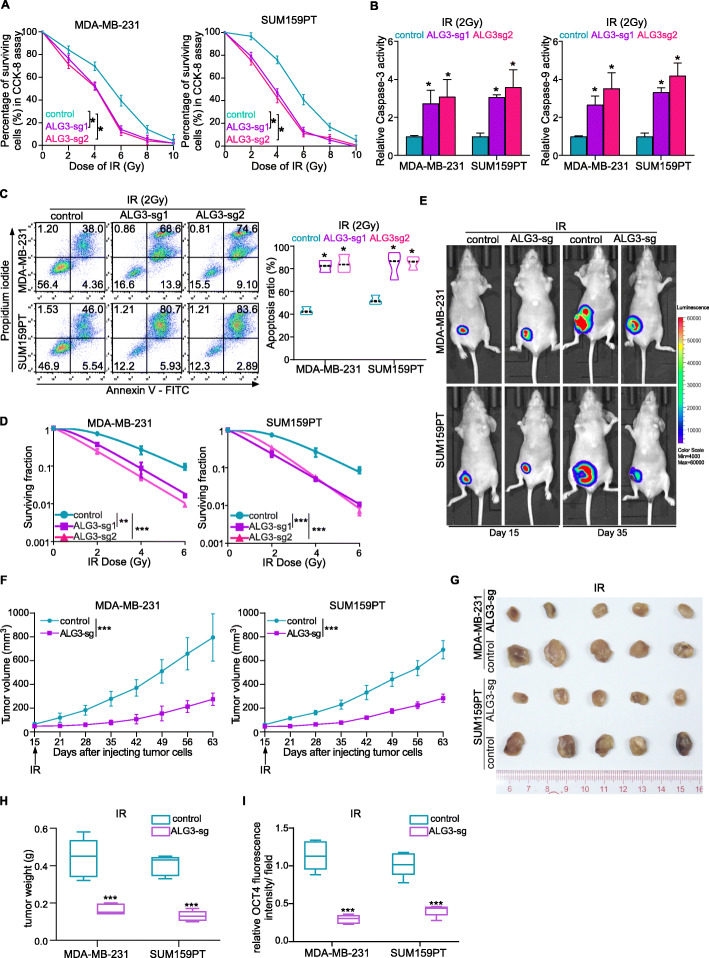
Down-regulation of ALG3 improves radiosensitivity. **a** Cell viability of SUM159PT and MDA-MB-231 irradiated with 0, 2, 4, 6, 8 and 10 Gy X-rays and cultured for 72 h were detected by CCK-8 assays. Data were analyzed by two-way ANOVA with Bonferroni’s post test. Each bar represents the mean ± SD of three independent experiments. **b** Caspase-3 and Caspase-9 activity in MDA-MB-231 and SUM159PT cells. Data were analyzed by one-way ANOVA with Tukey’s multiple comparison test. Each bar represents the mean ± SD of three independent experiments. **c** Apoptosis ratio of MDA-MB-231 and SUM159PT cells after treated with 2 Gy X-rays and cultured for 72 h was detected by flow cytometry. Data were analyzed by one-way ANOVA with Tukey’s multiple comparison test. Each bar represents the mean ± SD of three independent experiments. **d** The clonogenic survival fraction curve was fitted using a multitarget single-hit statistical model by colony assay. Data were analyzed by two-way ANOVA with Bonferroni’s post test. **e** Representative fluorescence graph of tumor-bearing mice with different ALG3 expression, which were treated with 2 Gy X ray. **f** Tumor growth rate was significantly decreased in ALG3-knocked out cells after radiation treatment. Data were analyzed by two-way ANOVA with Bonferroni’s post test. **g** Representative pictures of tumors from ALG3-sg + IR and control + IR mice were as shown. **h** The tumor weight of the ALG3-sg group after radiation treatment and the tumor weight of the control group after radiation treatment were calculated and shown. Data were analyzed by Student’s t-test. **i** The fluorescence intensity of OCT4 in ALG3-sg cells relative to that in control cells were measured. Data were analyzed by Student’s t-test. *P < 0.05, **P < 0.01, *** *P* < 0.001

To further examine the effect of downregulation of ALG3 on radiosensitivity in relative radioresistant cell lines, MDA-MB-231 and SUM159PT, we established stable ALG3-knocked out cell lines (MDA-MB-231-sg1, MDA-MB-231-sg2, SUM159PT-sg1, SUM159PT-sg2) (Supplementary Figure S3A). Compared with control groups, radiation treatment significantly reduced survival rates in the ALG3 knocked-out group, and increased the activity of caspase-9 and caspase-3 under the radiation treatment (*P* < 0.0001) (Fig. 3a and b). Apoptotic ratio was remarkably upregulated in ALG3-sg cells (ALG3 knocked out) under the 2 Gy radiation treatment compared with the control group (Fig. 3c). To further evaluate the radiosensitivity of tumor cells, we also performed colony formation assays with MDA-MB-231 and SUM159PT cell lines (Supplementary Figure S3B) and clonogenic survival fraction curves were generated basing on multitarget-single hit model (Fig. 3d). The mean surviving fraction and the *p* values between control group and ALG3-sg group were shown in Supplementary Table S5–6. The mean radiobiological parameters, N, D_0_, D_q_ and SF2 were calculated according to the curves (Table 2). The values of N, D_q_, D_0_, SF2 were higher in the control group than that in the ALG3 knocked-out (KO) groups, which indicated that significantly greater the dose was required to kill cells in control group. We further used orthotopic xenograft tumor models to determine the role of silencing ALG3 in sensitivity of breast cancer cells to radiation in vivo. ALG3-sg cells and the control cells were injected subcutaneously into the flanks of female mice. The radiation treatment strategy was the same as described above. Compared with control group, fluorescence intensity was significantly reduced in the ALG3-sg group after radiation treatment (Fig. 3e). Consistently, tumor volumes and weight in the ALG3-sg group were significantly suppressed after radiation treatment (Fig. 3f-3h). Moreover, we found that silencing ALG3 reduced OCT4 intensity in the tumor tissues from the mice (Fig. 3i). The IHC staining showed that ALG3 expression was reduced in more ALG3-sg tumor tissues than relative to in the vector tumors tissues at the end of the experiment (Supplementary Figure S3C). As shown in Supplementary Figure S3D-E, compared with control group, the expression of Ki67 was significantly reduced and caspase-3 activity was significantly increased in the ALG3-sg group after radiation treatment, which further proved that both the apoptosis and proliferation process were involved in ALG3 induced radioresistance. Collectively, downregulation of ALG3 sensitized breast cancer cells to radiation in vitro and in vitro.

### ALG3 promotes CSC-like traits in breast cancer

**Fig. 4:**
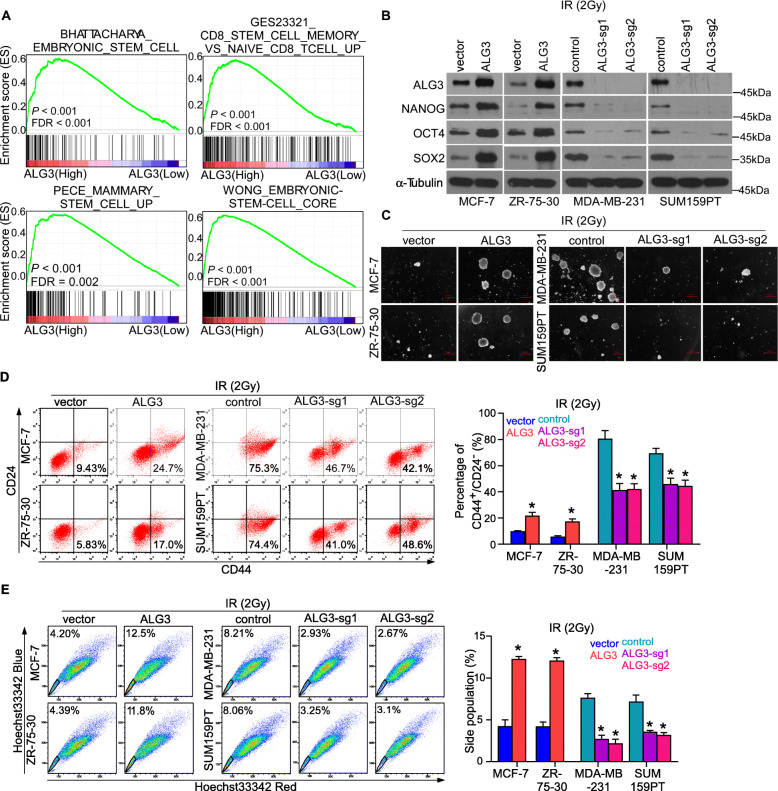
ALG3 promotes cancer stem-like traits of breast cancer cells. **a** Four different GSEA gene sets showed that ALG3 expression was positively correlated with CSC-associated gene signatures in a published breast cancer dataset. **b** NANOG, OCT4, and SOX2 expression were up-regulated in ALG3-tansduced cells and down-regulated in ALG3-knocked out cells. α-tubulin was detected as a loading control in the Western blot. **c** Sphere formation capacity was shown in four breast cancer cell lines. **d** Representative and statistic graph of flow cytometry analysis of MCF-7, ZR-75-30, MDA-MB-231, and SUM159PT cell lines stained with antibodies against cell surface markers CD44 and CD24. Data were analyzed by Student’s t-test and one-way ANOVA with Tukey’s multiple comparison test. Each bar represents the mean ± SD of three independent experiments. **e** Representative and statistic graph of flow cytometry detecting the percentage of side population cells of each sample. Data were analyzed by Student’s t-test and one-way ANOVA with Tukey’s multiple comparison test. Each bar represents the mean ± SD of three independent experiments. *P < 0.05, **P < 0.01

Previous studies have reported that cancer stem cells significantly contribute to the development of radioresistance in cancer scenario [47], and OCT4 intensity was significantly upregulated in ALG3-overexpressing tumor tissues from the mice (Fig. 2j). Therefore, we further investigated whether ALG3 could promote breast cancer stem-like traits. Gene set enrichment analysis (GSEA) analysis indicated that ALG3 expression is positively correlated with multiple stem cell-associated gene set signatures (Fig. 4a). Then, the expression of ALG3, NANOG, OCT4 and SOX2 were detected by Western blotting. As shown in Fig. 4b, downregulation of ALG3 reduced, while upregulation of ALG3 increased NANOG, OCT4 and SOX2 expression in breast cancer cells under radiation treatment. To further provide a proof that cancer stem like phenotype cells are the ones that grow and give rise to larger tumors, we performed the correlation analysis between ALG3 and cell cycle arrest associated genes, and found that CCNB1 (cyclin B1), CCNB2 (cyclin B2), and CDK4 were the most related genes. And the results of WB showed that ALG3 overexpressing cells had a high level of cyclin B1, cyclin B2, and CDK4 expression after radiation treatment (Supplementary Figure S4A-B). The upregulated of cyclin B1, cyclin B2, and CDK4 indicated the radiation treatment stimulates the subpopulation of cancer-stem-like cells cell cycle progression and proliferation. To verify if ALG3 regulates cancer stem-like function, sphere formation assay was further performed, and the results showed that ALG3-transduced cells exhibited significant more and larger spheres compared with vector control group in the MCF-7 and ZR-75-30 cells under radiation treatment. Conversely, ALG3-sg cells impaired sphere formation ability in MDA-MB-231 and SUM159PT cells (Fig. 4c). The capacity of secondary sphere formation is a hallmark of the stem cell property of self-renewal, and the rate of secondary sphere formation was calculated and presented in Supplementary Figure S4C-D. As the CD44^+^CD24^−^ subpopulation is considered to exhibited cancer stem-like traits in breast cancer [48], we further investigated whether ALG3 affects the percentage of CD44^+^CD24^−^breast cancer cells. As shown in Fig. 4d, we found that the CD44^+^CD24^−^ subpopulation was enriched in MDA-MB-231 and SUM159PT cells (high ALG3 expression) compared with ZR-75-30 and MCF-7 cells. Moreover, compared with control group of SUM159PT and MDA-MB-231 cells, CD44^+^CD24^−^ subpopulation was significantly decreased in ALG3-sg groups; compared with vector group of ZR-75-30 and MCF-7 cells, the CD44^+^CD24^−^ subpopulation was significantly increased in ALG3-transduced group. Considering that side population (SP) assay was always performed to detect cells with stem cell-like traits. We further performed SP assay and found that upregulation of ALG3 increased the proportion of side population cells, whereas downregulation of ALG3 had the opposite effect (Fig. 4e). Collectively, these results demonstrate that ALG3 promotes CSC-like traits in breast cancer.

### Silencing ALG3 inhibits activity of TGF-β signaling by reducing TGFBR2 glycosylation

**Fig. 5:**
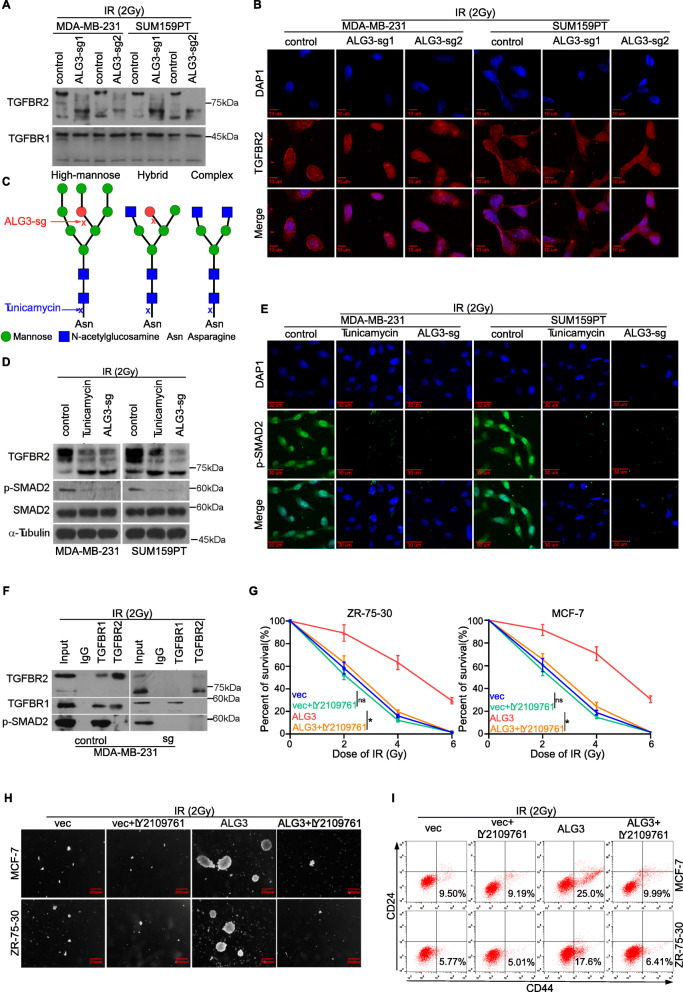
ALG3 enhances radioresistance via regulation of TGFBR2 glycosylation. (**a**) Downshift of TGFBR2 bands in ALG3-sg cells was detected by Western blot. But not TGFBR1 bands (**b**) Representative immunofluorescence images of TGFBR2 expression level in cytoplasmic and membrane fractions. (**c**) A schematic model of different subtypes of N-glycans. The round spots are mannose, the square ones are acetylglucosamine, and the red spot is the initial of the N-glycosylation site, which is initiated by ALG3. (**d**) TGFBR2 band shift could be seen in ALG3-sg cells or cells treated by tunicamycin. And downregulation of ALG3 reduced the expression level of p-SMAD2. (**e**) Representative immunofluorescence images of p-SMAD2 expression level in cytoplasmic and nuclear fractions. Nuclear translocation of p-SMAD2 was significantly decreased in ALG3-sg and tunicamycin treatment groups. (**f**) The co-immunoprecipitation between TGFBR1 and TGFBR2, TGFBR1 and p-SMAD2 could be detected in ALG3-control group, but not tunicamycin treatment, and ALG3-sg groups. (**g**) TGFBR2 inhibitor (LY2109761) in ALG3-transduced cells decreased the surviving fraction of breast cancer cells after radiation treatment, which were detected by CCK-8 assays. Data were analyzed by two-way ANOVA. Each bar represents the mean ± SD of three independent experiments. (**h**) Inhibition of TGFBR2 in ALG3-transduced cells decreased the number of colonies after radiation treatment. (**i**) Inhibition of TGFBR2 in ALG3-transduced cells decreased the proportion of CD44^+^CD24^−^ cells, which were detected by flow cytometry. “ns” no significance, *P < 0.05

Several lines of evidence have reported that multiple signaling pathways are closely associated with the maintenance of CSC-like traits, including Wnt [49, 50], TGF-β [51, 52] and PI3K/AKT [53], Hypoxia [54] and the antioxidant pathway [55], all of which have been reported to play important roles in radiation injury. To determine which pathway is the most relevant one affected by ALG3, a luciferase assay was conducted in SUM159PT and MDA-MB-231 cells. As shown in Supplementary Figure S5A-B, we found that downregulation of ALG3 reduced luciferase signal of smad-luc and TCF-1-luc, especially the smad-luc, suggesting that ALG3 promotes radioresistance and CSC-like traits probably by TGF-β signaling. In classical TGF-β pathway, N-glycosylation of TGF-β receptors has been reported to be indispensable for signal transduction, and ALG3 is associated with early N-glycans synthesis [28, 32]. Hence, we first examine whether ALG3 has an effect on TGF-β receptors’ glycosylation. As shown in Fig. 5a, we found that ALG3-sg significantly downshifted the TGFBR2 bands, but not the TGFBR1 bands (Fig. 5a). Interestingly, the total level of TGFBR2 was not obviously affected by ALG3-sg (Fig. 5a), implying that glycosylation of TGFBR2 may mediate the effect of ALG3 on activity of TGF-β signaling. It may be true that ALG3 mutation led to lower shift when glycosylated proteins are under-glycosylated [56], and under-glycosylated TGFBR2 was reported to exhibit bands lower shift in a range of 55 kDa–90 kDa in Western blot assay [26]. Furthermore, under-glycosylation of TGFBR2 reduced expression levels of TGFBR2 on membrane [57], which was supported by the immunofluorescence staining in our study (Fig. 5b), leading to the decreased activity of TGF-β signaling [57]. The glycosylation action sites of ALG3, as well as tunicamycin, an agent to block N-linked glycosylation, were displayed in Fig. 5c according to the previous studies [58]. ALG3 catalyzes the first mannosylation to the lipid-linked Man5GlcNAc2, which results in accumulation of high-mannose glycans (Fig. 5c, left) [34]. High mannose type of glycans has been reported significantly elevated in breast cancer cells and associated with poor prognosis [59]. Tunicamycin is widely used as a research tool to block N-linked glycosylation, which inhibits the enzymatic reactions between polyisoprenyl phosphate and UDP-GlcNAc or UDP-MurNAc-pentapeptide [60]. To verify whether the band shift was caused by a change in the glycans, tunicamycin was used as a positive control. As shown in Fig. 5d, the intensity of the lower TGFBR2 bands increased, leading to reduced p-smad2 expression in ALG3-sg cells, even to the level of tunicamycin treatment. Since nuclear p-smad2 level is responsible for genes transcription, immunofluorescence assay further performed to investigate the effect of ALG3 on nuclear transport of p-smad2. As shown in Fig. 5e, we found the nuclear translocation of p-smad2 was significantly suppressed in both tunicamycin and ALG3-sg groups (Fig. 5e). To further clarify how under-glycosylated TGFBR2 inactivates downstream pathway, we further performed a reciprocal immunoprecipitation and found that co-immunoprecipitation between TGFBR1 and TGFBR2, and TGFBR1 and p-smad2 could be detected in ALG3-control group (Fig. 5f). However, the co-immunoprecipitation was not observed in ALG3-sg group (Fig. 5f), indicating that downregulation of ALG3-induced under-glycosylated TGFBR2 disrupts the binding capacity with TGFBR1, further attenuating phosphorylation of smad2. Importantly, the expression levels of downstream target genes of TGF-β signaling were significantly reduced (red font) in both tunicamycin and ALG3-sg groups (Supplementary Figure S5C). Therefore, our results demonstrated that Silencing ALG3 inhibits activity of TGF-β signaling by disturbing TGFBR2 glycosylation.

### TGFBR2 is essential for ALG3-induced radioresistance and CSC-like traits

To demonstrate whether TGFBR2 is necessary for ALG3-induced radioresistance, we inhibited TGFBR2 activity in MCF-7 and ZR-75-30 cells with LY2109761 [61]. We found that ALG3-overexpressing increased the surviving fraction, while inhibition of TGFBR2 using LY2109761 in ALG3-transduced cells dramatically decreased the surviving fraction under radiation treatment (Fig. 5g). However, only inhibiting TGFBR2 in the vector breast cancer cells (vector +LY2109761) exhibited little effect on the surviving fraction compared with the vector breast cancer cells (Fig. 5g). This may be explained by the finding that the baseline expression of ALG3 is relatively low in MCF-7 and ZR-75-30 cells. Consistently, LY2109761 increased apoptotic ratio after radiation treatment in ALG3-overexpressing group in the MCF-7 and ZR-75-30 cells (Supplementary Figure S5D-E). In terms of CSC-like traits, LY2109761 abrogated the stimulatory effect of ALG3 on sphere formation ability (Fig. 5h and S5F), proportion of CD44^+^CD24^−^ cells in ALG3-overexpressing cells (Fig. 5i and S5G). Collectively, these results indicate that TGFBR2 mediates ALG3-induced radioresistance and CSC-like traits.

### Radiation therapy improves poor local recurrence-free survival in breast cancer patients with downexpression of ALG3

**Fig. 6:**
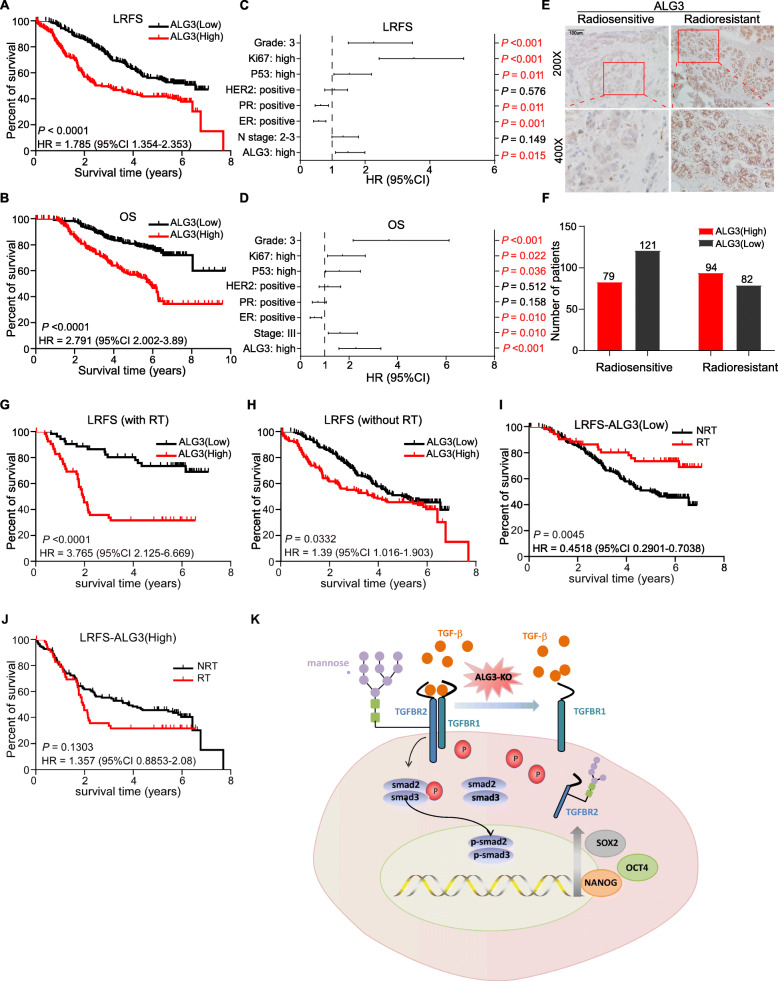
The association of ALG3 expression with prognosis in breast cancer patients. **a, b** LRFS and OS of breast cancer patients, stratified by high and low ALG3 expression, were analyzed by Kaplan-Meier analysis with log-rank test. **c, d** Multivariate Cox regression analysis to evaluate the significance of the association between ALG3 expression and prognosis were performed. **e** Representative images of ALG3 expression level detected by IHC were shown. **f** Statistical graph of ALG3 expression in patients who relapsed in 5 years or not was shown. **g** LRFS of breast cancer patients who underwent radiotherapy, stratified by ALG3 expression, was analyzed by Kaplan-Meier analysis with log-rank test. **h** LRFS of breast cancer patients who have not received radiotherapy, stratified by ALG3 expression, was analyzed by Kaplan-Meier analysis with log-rank test. **i** LRFS of breast cancer patients with ALG3 low-expression, stratified by undergoing radiotherapy or not, was analyzed by Kaplan-Meier analysis with log-rank test. **j** LRFS of breast cancer patients with ALG3 high-expression, stratified by undergoing radiotherapy or not, was analyzed by Kaplan-Meier analysis with log-rank test. **k** A schematic model of how ALG3 regulating TGF-β pathway was shown

Finally, the clinical correlation between ALG3 expression and prognosis of breast cancer patients was further analyzed in 376 paraffin-embedded breast cancer tissues using IHC staining. Table 3 showed the patients’ baseline clinical information and Table 4 showed the association of ALG3 expression with other clinicopathologic features. Kaplan-Meier survival curves of LRFS and OS were plotted according to high and low ALG3 expression level. As shown in Fig. 6a and b, patients with overexpression of ALG3 had a poorer prognosis compared to patients with relatively low-expression of ALG3. The analysis results of two independent publicly available datasets from Kaplan-Meier Plotter and TCGA showed that high level of ALG3 predicted shorter recurrence free survival (RFS), post progression free survival (PPS) and OS (Supplementary Figure S6A-E). Then, Univariate and Multivariate analysis were performed to assess the independent predictive ability of ALG3 in breast cancer patients. Univariate analysis showed that ALG3 expression, N-stage, estrogen/progesterone receptor (ER/PR), HER2, P53, Ki67 and grade are prognostic factors of LRFS. Meanwhile, ALG3 expression, TNM stage, N-stage, ER/PR, HER2, P53, Ki67 and grade were shown to be prognostic factors of OS (Table 5). Multivariate analysis showed that ALG3 expression, estrogen/progesterone receptor (ER/PR), P53, Ki67 and grade are independent prognostic factors of LRFS, whereas ALG3 expression, TNM stage, ER, P53, Ki67 and grade are independent prognostic factors of OS (Fig. 6c and d). As shown in Fig. 6e, ALG3 expression was dramatically upregulated in radioresistant human-breast cancer tissue compared with radiosensitive breast cancer tissues. Furthermore, the number of patients with ALG3 high-expression was higher than that with ALG3 low-expression in group who relapsed in 5 years, whereas the number of patients with ALG3 high-expression was less than that with ALG3 low-expression in group who did not relapse in 5 years (Fig. 6f). Then, we further analyzed the clinical significance of ALG3 in LRFS in breast cancer patients based on radiotherapy treatment history. As shown in Fig. 6g and h, overexpression of ALG3 predicted poorer LRFS no matter whether the patients receive radiation therapy. The therapeutic efficacy of radiation in breast cancer patients was further investigated based on the ALG3 expression levels, and the results showed that radiation therapy significantly improved LRFS in breast cancer patients with low ALG3 levels (Fig. 6i). However, radiation therapy had no significant effect on LRFS in breast cancer patients with high ALG3 levels (Fig. 6j). This finding supported the notion that in breast cancer patients with low ALG3 levels, radiation might be useful as an adjunctive therapy to delay the recurrence of breast cancer. However, for those with high ALG3 levels, radiation therapy does not show significant efficacy on LRFS.

**Table 3:** Characteristics of breast cancer patients

Characteristics	No. of patients (%)
**Age (years)**	
Median	47
Range	25–84
**Stage (AJCC**)	
I/II	188 (50)
III/IV	188 (50)
**Histological grade**	
1	108 (29)
2	179 (48)
3	89 (23)
Missing	0 (0)
**ER**	
Negative	169 (45)
Positive	207 (55)
Missing	0 (0)
**PR**	
Negative	124 (33)
Positive	252 (67)
Missing	0 (0)
**HER2**	
Negative	211 (56)
Positive	165 (44)
Missing	0
**Luminal**	288 (77)
**HER-2 subtypes**	53 (14)
**TNBC**	35 (9)
**Ki-67**	
Low expression^a^	281 (75)
High expression^b^	88 (23)
Missing	7 (2)
**Mutational P53**	
Low expression^a^	196 (52)
High expression^b^	165 (44)
Missing	15 (4)
**VEGF**	
Low expression^a^	302 (80)
High expression^b^	64 (17)
Missing	10 (3)
**Radiotherapy**	
Yes	91 (24)
No	285 (76)
**Chemotherapy**	
Yes	367 (98)
No	9 (2)

^a^Low expression represented -, +, ++ staining index in IHC examination

^b^High expression represented +++, ++++ staining index in IHC examination

**Table 4:** Association between ALG3 expression and other clinicopathologic

Features of breast cancer	No. of patients	ALG3 expression		***p*** values
		Low expression	High expression	
**Age**				0.920^†^
≤ 50	225	121	104	
> 50	151	82	69	
**Stage (AJCC)**				0.010^#^
I ~ II	188	114	74	
III ~ IV	188	89	99	
**T**				0.017^#^
T1 ~ T2	290	164	126	
T3 ~ T4	86	39	47	
**N**				0.028^#^
N0 ~ N2	205	123	82	
N2 ~ N3	171	80	91	
**Histological grade**				0.068^#^
1	108	69	39	
2	179	88	91	
3	89	46	43	
**ER**				0.011^†^
Negative	169	79	90	
Positive	207	124	83	
Missing	0			
**PR**				0.049^†^
Negative	124	58	66	
Positive	252	145	107	
Missing	0			
**HER2**				0.205^†^
Negative	211	158	124	
Positive	165	45	49	
Missing	0			
**Ki67**				0.001^#^
Low expression^a^	281	161	120	
High expression^b^	88	42	46	
Missing	7			
**Mutational p53**				0.001^#^
Low expression^a^	196	133	90	
High expression^b^	165	64	74	
Missing	15			
**VEGF**				0.167^#^
Low expression^a^	302	165	147	
High expression^b^	64	38	26	
Missing	10			

†*p* -values and #*p* -values were calculated with the chi-square test and continuity correlation, respectively

^a^Low expression represented -, +, ++ staining index in IHC examination

^b^High expression represented +++, ++++ staining index in IHC examination

**Table 5:** Univariate analyses of prognostic factors in breast cancer using a Cox regression model

		Univariate analyses (OS)		Univariate analyses (LRFS)	
	***No***. of patients	***p***	EXP(B) (SE)	***p***	EXP(B) (SE)
**ALG3**		< 0.001	2.702 (0.180)	0.013	1.739 (0.147)
Low expression	203				
High expression	173				
**Age**		0.339	1.181 (1.174)	0.350	0.8698 (0.151)
≤ 50	225				
> 50	151				
**Stage (AJCC)**		< 0.001	1.851 (0.176)	0.352	1.146 (0.146)
I ~ II	188				
III	188				
**T**		0.098	1.384 (0.196)	0.620	1.091 (0.175)
T1 ~ T2	290				
T3 ~ T4	86				
**N**		< 0.001	1.991 (0.174)	0.022	1.397 (0.146)
N0 ~ N2	205				
N2 ~ N3	171				
**Histological grade**					
1(indicator)	108				
2	179	0.021	1.644 (0.216)	0.065	0.917 (0.167)
3	89	< 0.001	3.301 (0.247)	0.047	1.483 (0.198)
**ER**		< 0.001	0.444 (0.1745)	< 0.001	0.445 (0.148)
Negative	169				
Positive	207				
Missing	0				
**PR**		< 0.001	0.496 (0.174)	< 0.001	0.430 (0.147)
Negative	124				
Positive	252				
Missing	0				
**HER2**		0.008	1.582 (0.172)	< 0.001	1.729 (0.147)
Negative	211				
Positive	165				
Missing	0				
**Ki67**		< 0.001	2.006 (0.180)	< 0.001	3.547 (0.149)
Low expression^a^	281				
High expression^b^	88				
Missing	7				
**Mutational P53**		< 0.001	2.139 (0.177)	< 0.001	2.695 (0.150)
Low expression^a^	196				
High expression^b^	165				
Missing	15				
**VEGF**		0.853	1.042 (0.222)	0.128	1.315 (0.180)
Low expression^a^	302				
High expression^b^	64				
Missing	10				

^a^Low expression represented -, +, ++ staining index in IHC examination

^b^High expression represented +++, ++++ staining index in IHC examination

To find out if ALG3 have a stronger predictive ability in a particular tumor type, we perform the subgroups analysis based on tumor type (Supplementary Figure S7A-B). Considering the small sample size in TNBC group, the Kaplan–Meier survival curves suggested that ALG3 was a predictive factor independent of tumor types, but it had a better discrimination in TNBC patients. Further, we evaluated the prognostic value for radiosensitivity of ALG3 in hormone receptors positive, and hormone receptors negative with or without radiotherapy (Supplementary Figure S7C-D). Survival curves showed that higher expression of ALG3 in patients who received radiotherapy were positively correlated with a worse survival outcome independent the hormone receptors status. Similarly, we then evaluated the prognostic value of radiotherapy in hormone receptor positive, and hormone receptor negative stratified by ALG3 expression (Supplementary Figure S7E). And the results indicated that radiotherapy tended to have little effect for patients with ALG3 overexpression.

TP53 mutation was demonstrated contributing to radioresistance in several researches, which occurred in about 50% sporadic breast cancer patients, especially TNBC patients [62]. The mutational p53 protein was detectable by IHC, which is a routine examination in clinic. Thus, we analyzed the predictive value of ALG3 expression based on p53 status. Table 4 showed the ALG3 expression was positively associated with p53 status. The Kaplan–Meier survival curves suggested that ALG3 was a predictive factor independent of p53 status (Supplementary Figure S7F). Moreover, no matter in the situation of ALG3 overexpression or p53 positive, radiotherapy did not improve outcome for improving outcomes in breast cancer (Supplementary Figure S7G-H). The number of patients in each subgroup was shown in Table 3 and Supplementary Table S7.

Collectively, our results indicate that overexpression of ALG3 is correlated with poor survival in breast cancer patients. For breast cancer patients with low ALG3 levels, radiation therapy is beneficial to improve prognosis as a therapeutic strategy.

Together, our study demonstrated that ALG3 overexpression contributed to the radioresistance of breast cancer though regulating glycosylation of TGF-β receptor II (Fig. 6k).

## Discussion

Radiotherapy is playing an increasingly important role in breast cancer treatment with advancement of technology. However, the prognosis after RT varies greatly among breast cancer patients [63], which may be significantly associated with the development of radioresistance. Although a lot of research effort has been devoted to discover predictive markers of radioresistance in breast cancer [64–66], but no reliable markers have been widely used in clinical practice. A reliable predictive maker of radiosensitivity can effectively guide personalized treatment decisions. Currently, glycosylated proteins have become one of the most common cancer biomarkers in the clinic, such as alpha-fetoprotein (AFP) for hepatocellular carcinoma [67], carcinoembryonic antigen (CEA) for colon cancer [68] and prostate specific antigen (PSA) for prostate cancer [69]. However, glycoprotein-related predictive maker for breast cancer, especially for radioresistant breast cancer, remains blank. In this study, we found that ALG3 was dramatically upregulated in radioresistance breast cancer tissues, which predicted a high rate of recurrence as well as poor mortality in breast cancer patients. Gain and loss of function experiments demonstrated that upregulating ALG3 promoted, while silencing ALG3 improved resistance of breast cancer cells to radiation therapy in vitro and in vivo. Importantly, our results found that radiation therapy significantly improved LRFS in breast cancer patients with low ALG3 levels, but it had no significant effect on LRFS in breast cancer patients with high ALG3 levels. Collectively, our results indicate that ALG3 may serve as a potential marker to predict radiosensitivity, as well as a radiation sensitizer to improve radioresistance in breast cancer patients with high ALG3 levels. For patients with low ALG3 levels, radiation remains an effective mainstay therapy to prevent early recurrence in breast cancer.

Cancer stem-like traits have been extensively demonstrated to be associated with radioresistance, which significantly contributes to the radioresistance and early relapse of cancers [70]. The biological function of CSC-like trait promoting radioresistance is implicated in DNA damage repair, hypoxia and cell cycle arrest [46]. Firstly, cancer-stem like cells spend most of their time in non-dividing G0 cell cycle state, and therefore, are resistant to IR. IR is a cell cycle-dependent treatment which are more effective against rapidly proliferating cells, particularly those cells in the mitotic phase [71]. Second, cancer-stem like cells exhibit greatly enhanced DNA damage repair ability compared with ordinary tumor cells, which is responsible for reducing DNA-damage induces apoptosis or necrosis after radiation treatment [72]. Finally, hypoxic microenvironment has been demonstrated playing an important role in CSC induce radioresistance [73], because that oxygen is essential for cytotoxic reactive oxygen species (ROS) production to damage tumor cells after radiation treatment [74]. Therefore, CSC-like traits were considered as important contributor to radioresistance [75]. In this study, we found ALG3 increased expression level of several crucial CSC markers, including Nanog, OCT4 and SOX2, enhanced sphere formation ability and increased the proportion of CD44^+^CD24^−^ cells. By contrast, silencing ALG3 dramatically suppressed cancer stemness in breast cancer cells. These findings indicate that ALG3 functions as a pivotal regulator for cancer stem-like traits in breast cancer, which further promotes the resistance of breast cancer cells to radiation therapy.

Cancer-stem like traits can be controlled by multiple manners, in which post-transcriptional modifications, especially glycosylation, has been reported to significantly contribute to cancer-stem like traits in a variety of cancers [15, 76, 77]. Glycosylation is a common posttranslational modification on membrane-associated and secreted proteins. Because of their special cell-surface and extracellular position, glycans are of pivotal importance in controlling cell-cell communication, signal transduction and receptor activation due to cell surface glycans being essential for cellular receipts of signals from outside [78]. Several key growth factors, such as EGF, hepatocyte growth factor (HGF), vascular endothelial growth factor (VEGF) and TGF-β play important role in glycosylation of the receptors, which further modulate the sensitivity of the receptors to ligands, the efficacy of signal transduction and cancer progression [78]. Among these, TGF-β pathway is regarded to be one of the most important pathways that contribute to cancer stemness in the different tumor entities including breast cancer [10, 79], since there are several important glycosylated proteins in TGF-β pathways, such as TGF-β, TGFBRs and smad. Previous studies have reported that some glycosyltransferases and glycosidases are associated with the phosphorylation of TGF-β receptors of TGF-β signaling, such as fucosyltransferase 8 (FUT 8) in lung cancer [80], sialylation in colon cancer [81] N-acetylglucosaminyl-transferase V (MGAT5, 37] and α2,3 sialytransferase 5,GM3 synthase (ST3GAL5) [26]. However, the effect of mannosyltransferase on cellular receipts has been poorly understood. There are mainly three major types of N-linked oligosaccharides: oligomannosidic (high mannose), hybrid and complex type glycan-structures [82]. Both complex type and oligomannosidic type are required for the successful cell surface transportation of TGFBR2 [26]. ALG family represents an important group of mannosyltransferase. For example, ALG3 has alpha-1,3-mannosyltransferase activity and initiates luminal oligosaccharide biosynthesis [83], which is essential for N-linked protein glycosylation [36] and ALG3 aberration results in loss of mannose in the oligosaccharide chain [84]. Recently, ALG3 has been reported to promote chemotherapy resistance by inducing mannosylation in acute myeloid leukemia [34]. However, the biological role of ALG3-induced glycosylation in radioresistant breast cancer remains largely unknown. In this study, we found that silencing ALG3 significantly reduced glycosylation of TGFBR2, as demonstrated by the lower shift bands of TGFBR2 in Western blot. Importantly, downregulating ALG3-induced under-glycosylation disrupts the binding capacity of TGFBR2 with TGFBR1, further attenuating phosphorylation of smad2 and inactivation of TGF-β signaling, which ultimately inhibits cancer cells stem-like traits and increased radiosensitivity in breast cancer cells. Furthermore, specific TGFBR2 inhibitor (LY2109761) differentially abrogated ALG3 overexpression-induced radioresistance and CSC-like traits. Collectively, our results indicate that ALG3 promotes radioresistance and CSC-like traits by activating TGF-β signaling dependent on glycosylation of TGFBR2 in breast cancer.

In our study, we performed the subgroup analysis by Kaplan-Meier survival curves to evaluate the predictive value of ALG3 in different tumor types and TP53 status of breast cancer. Supplementary Fig. S7A, B and F showed that ALG3 is a strong biomarker irrespective of tumor subtypes and TP53 status. Although *p* values were larger than 0.05 in HER-2 and TNBC subgroups due to the small sample size, the trend is consistent. Supplementary Fig. S7C-D showed that an obvious trend was observable in Luminal &RT, Non-luminal &RT, and Non-luminal &NRT subgroups, but not in Luminal &NRT. Possibly because of the good prognosis of luminal subtype. RT would further improve the outcome of ALG3(low) subgroup but not ALG3(high) subgroup. Luminal subtype and the ALG3(Low) were both positive factors for prognosis and Supplementary Fig. S7E showed that RT induced improvement of outcome was only observable in the subgroup Luminal & ALG3(Low). Considering that we could still see a trend in Non-luminal & ALG3(low) subgroup, we assumed that RT was also a proper treatment for Non-luminal& ALG3(low) subgroup. Fig. S7H showed a trend for the subgroup TP53(−) & ALG3(Low) but it was not significant for outcome after RT in the other subgroups, which was consistent with that both TP53 mutation [85] and ALG3 overexpression would significantly influence the radiosensitivity of breast cancer. And patients even seemed to do worse after RT in TP53(−) & ALG3(High) subgroup and Luminal & ALG3(High) subgroup, which suggested that there might be some interactions between ALG3 associated biological behavior and RT, and further demonstrated that patients with ALG3 low-expression were suitable for radiotherapy. ALG3 appeared to be a stronger biomarker in the TP53(+) & RT subgroup than in the TP53(+) & NRT subgroup. And the lower *p*-value without RT in Fig. S7G may be associated with the three-fold higher number of patients in TP53(+) & NRT subgroup.

Due to the small sample size in many subgroups, the predictive value of ALG3 based on different TP53 status and the tumor types needs to be investigated in a larger sample size in the future studies.

It is interesting that breast cancer cell lines with TP53 mutation tended to overexpress ALG3, except for HCC1937 and T47D cell lines, and the correlation analysis based on TCGA data indicated that breast cancer patients with TP53 mutation tended to overexpress ALG3. However, some patients with low expression of ALG3 exhibited detectable mutant p53, and some patients with high expression of ALG3 exhibited wild type p53 based on our patients’ information in our study, which does not support to the hypothesis that wild type p53 might inhibit expression of ALG3, while mutant p53 does not. And our results from subgroup analysis and multivariate analysis suggested that ALG3 predicted a worse prognosis independent of p53 status. Thus, the correlation between ALG3 and p53 status is may be a coincidence. Since mutant p53 has been reported to play an important role in radioresistance in several researches [85–87], we would like to investigate the interaction between ALG3 and mutant p53 in depth in the future.

## Conclusion

In summary, our results demonstrate that ALG3 overexpression promotes glycosylation of TGFBR2 and activates TGF-β signaling, which further promotes radioresistance and CSC-like traits in breast cancer. Thus, the critical findings of this current study present novel insights into the molecular mechanisms by which ALG3 promotes the resistance of breast cancer cells to radiation therapy, which will facilitate to improve radioresistance of breast cancer by targeting ALG3.

## Supplementary Information

**Additional file 1: Supplementary Figure 1.** (A) The distribution of patients with different tumor type and TP53 status was as shown (left); mRNA expression of ALG3 in subgroup with mutant p53 or wild-type p53 was as shown (right) Each bar represents the mean ± SD of the group. (B) TCGA dataset indicates ALG3 hyper-expresses in basal-like and HER2 positive cell lines. Each bar represents the mean ± SD of the group. (C) TCGA dataset indicates ALG3 tends to be overexpressed in cell lines with TP53 mutation, but not BRCA mutation. Each bar represents the mean ± SD of the group. (D) The correlation between relative RNA expression of ALG3 and the ratio of gray-scale (gray value of the target protein band / gray value of the-tubulin band) was shown. (E) TCGA dataset indicates gene gain or amplification contributes to ALG3 overexpression. Each bar represents the mean ± SD of the group. All data were analyzed by Student’s t-test. “ns” no significance, *P < 0.05, **P < 0.01, *** *P* < 0.001.

**Additional file 2: Supplementary Figure 2.** (A) ALG3 expression after transduction in MCF-7 and ZR-75-30 cell lines was confirmed by Western blot. (B) Representative images of colony assays, with cells exposed to 0,2,4,6 Gy X ray treatment were as shown. (C) Representative image of local radiation treatment was as shown. (D) Immunohistochemistry image of tumor tissues from ALG3-transduced mice depicts successful upregulation of ALG3. Original magnification, 200x and 400x. scale bar   =   50 μm. (E) Immunohistochemistry images showed that tumor tissues from ALG3-transduced mice overexpressing Ki67. Original magnification, 200x and 400x. scale bar  = 50 μm. (F) Cleaved caspase-3 activity was performed on tumor tissues from mice. *** *P* < 0.001.

**Additional file 3: Supplementary Figure 3.** (A) ALG3 expression after knocking out of ALG3 was confirmed in MDA-MB-231 and SUM159PT cell lines by Western blot. (B) Representative images of colony assays, with cells exposed to 0,2,4,6 Gy X ray treatment were as shown. (C) Immunohistochemistry image of tumor tissues from ALG3-sg mice depicts successful downregulation of ALG3. Original magnification, 200x and 400x. scale bar  =   50 μm. (D) Immunohistochemistry images showed that Ki67 expression of ALG3-sg mice was downregulated. Original magnification, 200x and 400x. scale bar  =   50 μm. (E) Cleaved caspase-3 activity was performed on tumor tissues from mice. *** *P* < 0.001.

**Additional file 4: Supplementary Figure 4.** (A) Analysis of correlation between ALG3 expression and cell cycle related genes based on TCGA database were performed. (B) Cyclin B1, cyclin B2, and CDK4 expression were up-regulated in ALG3-tansduced cells and down-regulated in ALG3-knocked out cells. α-Tubulin was detected as a loading control in Western blot. (C, D) Statistical graphs of secondary sphere formation assay in breast cancer cells were plotted. Each bar represents the mean ± SD of three independent experiments. All data were analyzed by Student’s t-test and one-way ANOVA with Tukey’s multiple comparison test. *P < 0.05.

**Additional file 5: Supplementary Figure 5.** (A, B) Radioresistance-associated pathways were examined by luciferase assay to find out the most influenced pathway after ALG3-knocked out. Each bar represents the mean ± SD of three independent experiments. All data were analyzed by one-way ANOVA with Tukey’s multiple comparison test. (C) mRNA expression level of several genes that regulated by TGF-β pathway in ALG3-sg cells or cells treated by tunicamycin compared with ALG3 control cells were detected by RT-PCR. GAPDH was detected as a loading control in RT-PCR. (D) Upregulation of ALG3 decreased the apoptosis ratio, whereas inhibition of TGFBR2 increased the apoptosis ratio of ALG3-transduced cells after radiation treatment, which were detected by flow cytometry. (E) Statistical graphs of apoptotic cells proportion in the vector, ALG3-vector +LY2109761(TGFBR2 inhibitor), ALG3-transduced, and ALG3-transduced +LY2109761 cells were plotted. Each bar represents the mean ± SD of three independent experiments. Data were analyzed by Student’s t-test. (F) Upregulation of ALG3 led to increasing the number of spheres, while inhibition of TGFBR2 decreased the number of spheres in ALG3-overexpressing cells, which were detected by secondary sphere assay. Each bar represents the mean ± SD of three independent experiments. Data were analyzed by Student’s t-test. (G) Upregulation of ALG3 led to increasing proportion of the CD44^+^CD24^−^subpopulation, whereas inhibition of TGFBR2 decreased proportion of the CD44^+^CD24^−^ subpopulation in ALG3-transduced cells. Each bar represents the mean ± SD of three independent experiments. All data were analyzed by Student’s t-test. *P < 0.05.

**Additional file 6: Supplementary Figure 6.** (A–C) Kaplan–Meier analysis using the public breast cancer data sets showed that the ALG3 overexpression indicates shorter RFS, PPS, and OS. Website, https://kmplot.com/analysis/. (D, E) Kaplan–Meier analysis showed that ALG3 overexpression indicates shorter RFS and OS in the TCGA database.

**Additional file 7: Supplementary Figure 7.** (A-B) LRFS and OS of different tumor type patients, stratified by high and low ALG3 expression, were analyzed by Kaplan-Meier analysis with log-rank test. (C-D) Kaplan–Meier curves of LRFS and OS were plotted to evaluate the predictive value of ALG3 in patients grouped by tumor type and receiving radiotherapy or not. (E) Kaplan–Meier curves of LRFS were plotted to evaluate if radiotherapy contribute to prognosis of patients with ALG3 overexpressing in different tumor type. (F) LRFS and OS of patients with different p53 status, stratified by high and low ALG3 expression, were analyzed by Kaplan-Meier analysis with log-rank test. (G) LRFS of patients with mutational p53 who have received radiotherapy or not, stratified by ALG3 expression was analyzed by Kaplan-Meier analysis with log-rank test. (H) Kaplan–Meier curves of LRFS curves were plotted to evaluate if radiotherapy contribute to prognosis of patients with ALG3 overexpressing or with mutational p53.

**Additional file 8: Table S1.** Molecular baseline characteristics of patients.

**Additional file 9: Table S2.** Association of ALG3 expression with TP53 status and tumor type.

**Additional file 10: Table S3.** The detail information of colony assay in MCF-7 cell line.

**Additional file 11: Table S4.** The detail information of colony assay in ZR-75-30 cell line.

**Additional file 12: Table S5.** The detail information of colony assay in MDA-MB-231 cell line.

**Additional file 13: Table S6.** The detail information of colony assay in SUM159PT cell line.

**Additional file 14: Table S7.** The number of patients in subgroups.

## Data Availability

The data that support the findings of this study are available from Research Data Deposit public platform but restrictions apply to the availability of these data, which were used under license for the current study, and so are not publicly available. Data are however available from the authors upon reasonable request and with permission of Research Data Deposit public platform.

## Abbreviations

ALG3: α-1,3-mannosyltransferase
Asn: Asparagine
BCSCs: Breast cancer stem cells
CSCs: Cancer stem cells
D_0_: Mean lethal dose
D_q_: Quasi—threshold dose
ER: Estrogen receptor
FUT: 8 Fucosyltransferase 8
GEO: Gene Expression Omnibus
GSEA: Gene Set Enrichment Analysis
HER2: Human epidermal growth factor receptor 2
IHC: Immunohistochemical
IR: Irradiation
KO: Knock out
LRFS: Local recurrence-free survival
luc,: Luciferase
MGAT5: N-acetylglucosaminyl-transferase V
NIR: Non-irradiated
NRT: Not received radiotherapy
N: Stage node metastasis
N: Extrapolated value
OS: Overall survival
PR: Progesterone receptor
RT-PCR: Real time polymerase chain reaction
RT: Radiotherapy
res: Radioresistant
RNAi: RNA interference
siRNA: Small interfering RNA
SF2: Survival fraction of 2 Gy
sen: Radiosensitive
ST3GAL5: Sialytransferase 5, GM3 synthase;
TCGA: The Cancer Genome Atlas
TGFBR1: Transforming growth factor beta receptor I
TGFBR2: Transforming growth factor beta receptor II
TGFBRs: Transforming growth factor beta receptors
