# Correction to: ALG3 contributes to stemness and radioresistance through regulating glycosylation of TGF-β receptor II in breast cancer

**DOI:** 10.1186/s13046-022-02322-4

**Published:** 2022-03-31

**Authors:** Xiaoqing Sun, Zhenyu He, Ling Guo, Caiqin Wang, Chuyong Lin, Liping Ye, Xiaoqing Wang, Yue Li, Meisongzhu Yang, Sailan Liu, Xin Hua, Wen Wen, Chao Lin, Zhiqing Long, Wenwen Zhang, Han Li, Yunting Jian, Ziyuan Zhu, Xianqiu Wu, Huanxin Lin

**Affiliations:** 1 Department of Medical Oncology, Sun Yat-sen University Cancer Center, Guangzhou, 510060 Guangdong People’s Republic of China; 2 Department of Radiotherapy, Sun Yat-sen University Cancer Center, Guangzhou, 510060 Guangdong People’s Republic of China; 3 Department of Nasopharyngeal Carcinoma, Sun Yat-sen University Cancer Center, Guangzhou, 510060 Guangdong People’s Republic of China; 4 Department of Medical Oncology, The Sixth Affiliated Hospital of Sun Yat-Sen University, Guangzhou, 510655 Guangdong People’s Republic of China; 5 Department of Experimental Research, State Key Laboratory of Oncology in Southern China, Sun Yat-sen University Cancer Center, Guangzhou, 510060 Guangdong China; 6 Department of Experimental Research, The Seventh Affiliated Hospital of Sun Yat-sen University, Shenzhen, 518107 Guangdong People’s Republic of China; 7 Department of Radiotherapy, Nanfang Hospital, Guangzhou, 510515 Guangdong People’s Republic of China; 8 Department of Physiology, Sun Yat-sen University, Guangzhou, 510080 Guangdong People’s Republic of China; 9 Department of Gynecological Oncology, Sun Yat-sen University Cancer Center, Guangzhou, 510060 Guangdong People’s Republic of China; 10 Department of General surgery, The Third Affiliated Hospital of Guangzhou Medical College, Guangzhou, 510150 Guangdong People’s Republic of China; 11 Clinical Experimental Center, Jiangmen Key Laboratory of Clinical Biobanks and Translational Research, Jiangmen Central Hospital, Affiliated Jiangmen Hospital of Sun Yat-sen University, Jiangmen, 529030 Guangdong People’s Republic of China

**Correction to: J Exp Clin Cancer Res 40, 149 (2021)**

**https://doi.org/10.1186/s13046-021-01932-8**

Following publication of the original article [1], the authors identified minor errors in Fig. 5, specifically:

- Fig. 5h: Incorrect sphere formation image used for ALG3+LY2109761 (4^th^ column, 1^st^ and 2^nd^ rows)
- Fig. 5i: The percentage of CD44+/CD24- in the image of flow cytometry was placed in the wrong quadrant

The corrected figure is given here. The corrections do not have any effect on the final conclusions of the paper. The original article has been corrected.

**Fig. 5:**
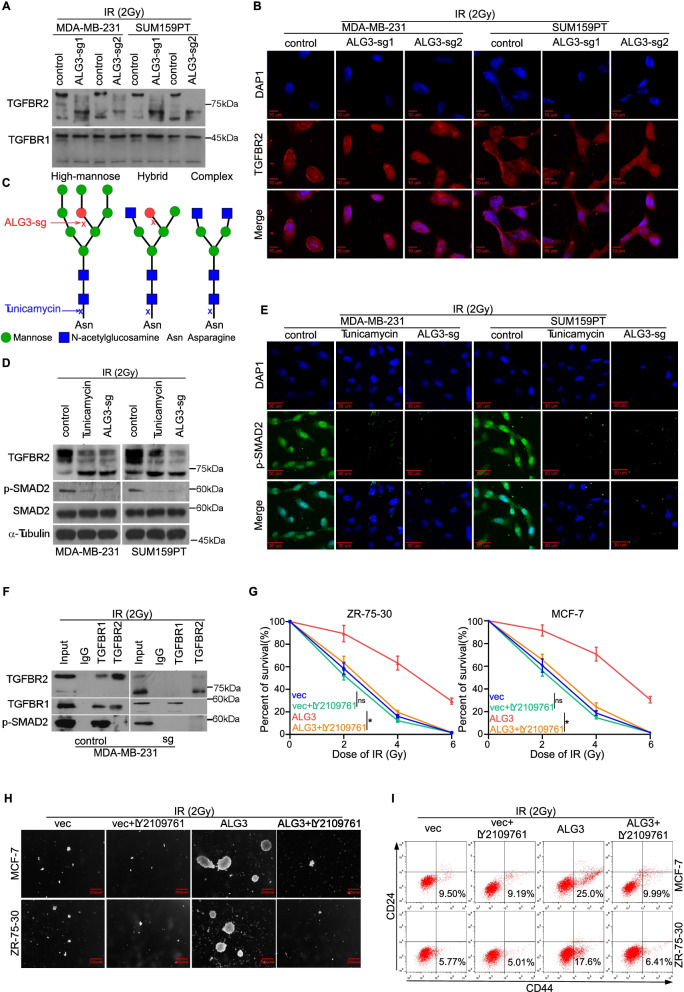
ALG3 enhances radioresistance via regulation of TGFBR2 glycosylation. **a** Downshift of TGFBR2 bands in ALG3-sg cells was detected by Western blot. But not TGFBR1 bands (**b**) Representative immunofluorescence images of TGFBR2 expression level in cytoplasmic and membrane fractions. **c** A schematic model of different subtypes of N-glycans. The round spots are mannose, the square ones are acetylglucosamine, and the red spot is the initial of the N-glycosylation site, which is initiated by ALG3. **d** TGFBR2 band shift could be seen in ALG3-sg cells or cells treated by tunicamycin. And downregulation of ALG3 reduced the expression level of p-SMAD2. **e** Representative immunofluorescence images of p-SMAD2 expression level in cytoplasmic and nuclear fractions. Nuclear translocation of p-SMAD2 was significantly decreased in ALG3-sg and tunicamycin treatment groups. **f** The co-immunoprecipitation between TGFBR1 and TGFBR2, TGFBR1 and p-SMAD2 could be detected in ALG3-control group, but not tunicamycin treatment, and ALG3-sg groups. **g** TGFBR2 inhibitor (LY2109761) in ALG3-transduced cells decreased the surviving fraction of breast cancer cells after radiation treatment, which were detected by CCK-8 assays. Data were analyzed by two-way ANOVA. Each bar represents the mean ± SD of three independent experiments. **h** Inhibition of TGFBR2 in ALG3-transduced cells decreased the number of colonies after radiation treatment. **i** Inhibition of TGFBR2 in ALG3-transduced cells decreased the proportion of CD44^+^CD24^−^ cells, which were detected by flow cytometry. “ns” no significance, *P < 0.05
