# Supplementary figures and images for: ALG3 contributes to stemness and radioresistance through regulating glycosylation of TGF-β receptor II in breast cancer
Source: J Exp Clin Cancer Res. 2021 Apr 30;40:149. doi: 10.1186/s13046-021-01932-8 (PMC8086123; doi:10.1186/s13046-021-01932-8)

**Fig. S1**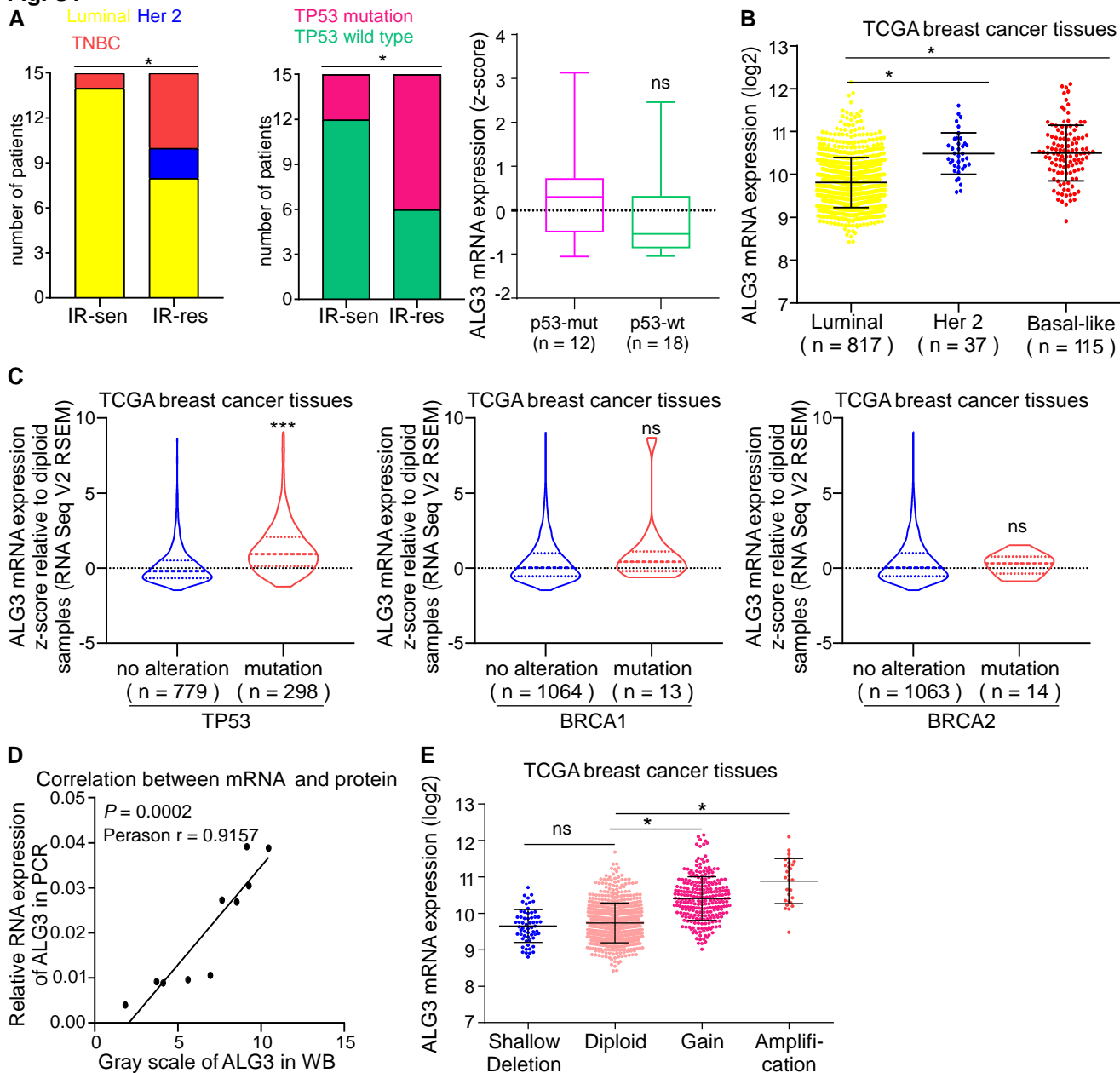

Supplement: Supplementary file 1 — Additional file 1: Supplementary Figure 1. (A) The distribution of patients with different tumor type and TP53 status was as shown (left); mRNA expression of ALG3 in subgroup with mutant p53 or wild-type p53 was as shown (right) Each bar represents the mean ± SD of the group. (B) TCGA dataset indicates ALG3 hyper-expresses in basal-like and HER2 positive cell lines. Each bar represents the mean ± SD of the group. (C) TCGA dataset indicates ALG3 tends to be overexpressed in cell lines with TP53 mutation, but not BRCA mutation. Each bar represents the mean ± SD of the group. (D) The correlation between relative RNA expression of ALG3 and the ratio of gray-scale (gray value of the target protein band / gray value of the-tubulin band) was shown. (E) TCGA dataset indicates gene gain or amplification contributes to ALG3 overexpression. Each bar represents the mean ± SD of the group. All data were analyzed by Student’s t-test. “ns” no significance, *P < 0.05, **P < 0.01, *** P < 0.001. [file 13046_2021_1932_MOESM1_ESM.pdf]

**Fig. S2**

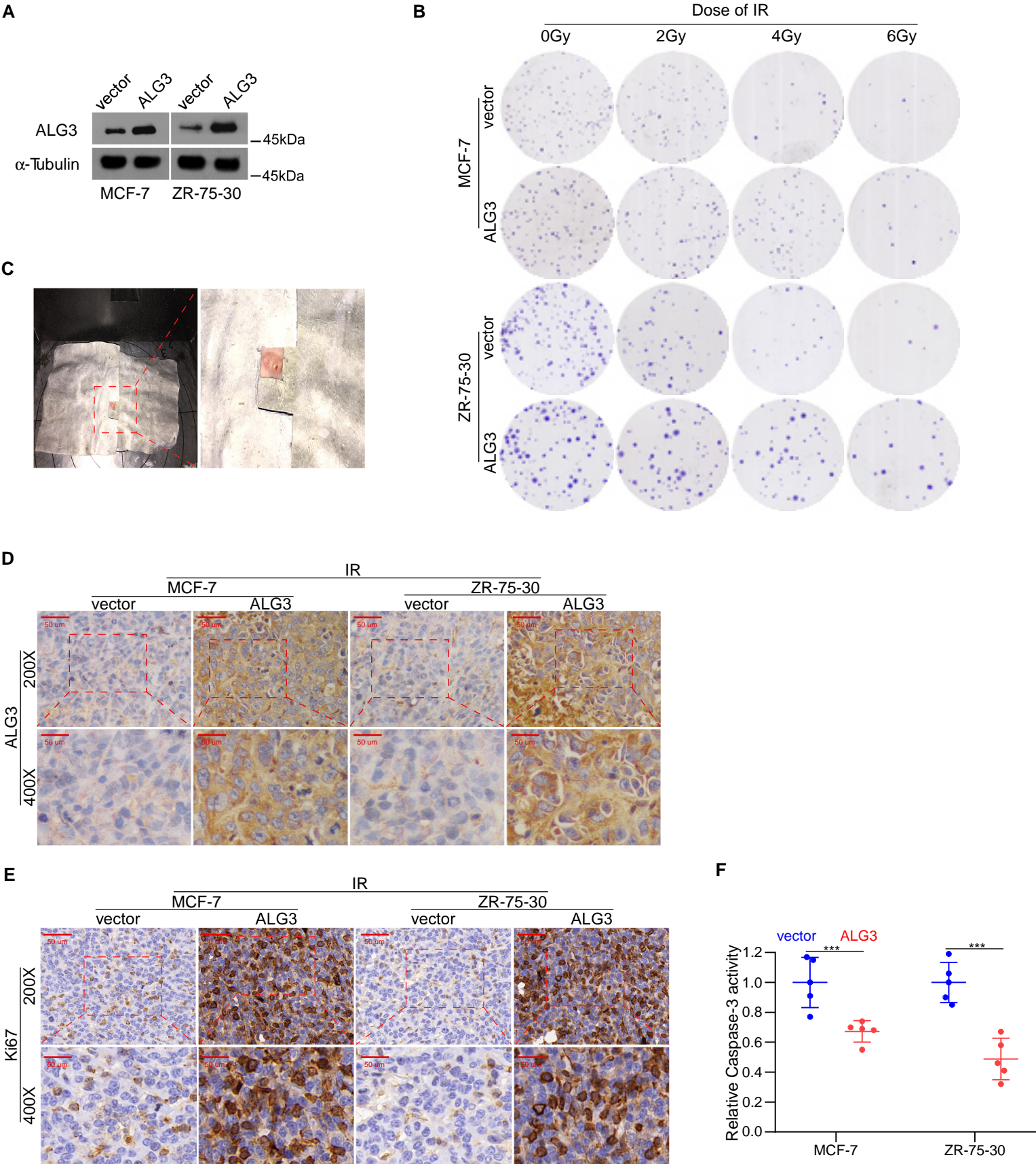

Supplement: Supplementary file 2 — Additional file 2: Supplementary Figure 2. (A) ALG3 expression after transduction in MCF-7 and ZR-75-30 cell lines was confirmed by Western blot. (B) Representative images of colony assays, with cells exposed to 0,2,4,6 Gy X ray treatment were as shown. (C) Representative image of local radiation treatment was as shown. (D) Immunohistochemistry image of tumor tissues from ALG3-transduced mice depicts successful upregulation of ALG3. Original magnification, 200x and 400x. scale bar = 50 μm. (E) Immunohistochemistry images showed that tumor tissues from ALG3-transduced mice overexpressing Ki67. Original magnification, 200x and 400x. scale bar = 50 μm. (F) Cleaved caspase-3 activity was performed on tumor tissues from mice. *** P < 0.001. [file 13046_2021_1932_MOESM2_ESM.pdf]

**Fig. S3****A**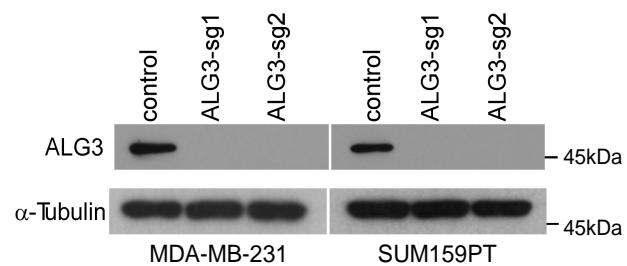**B**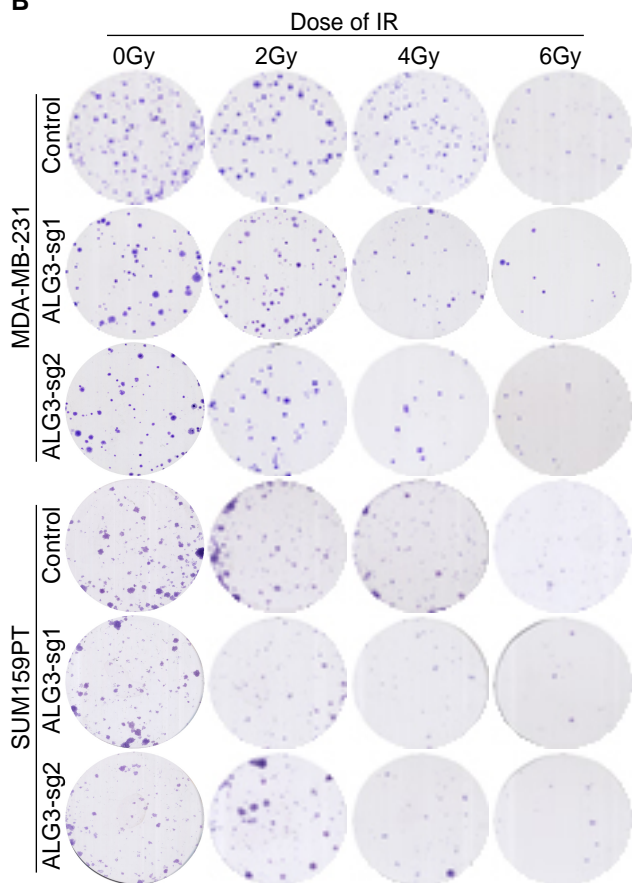**C**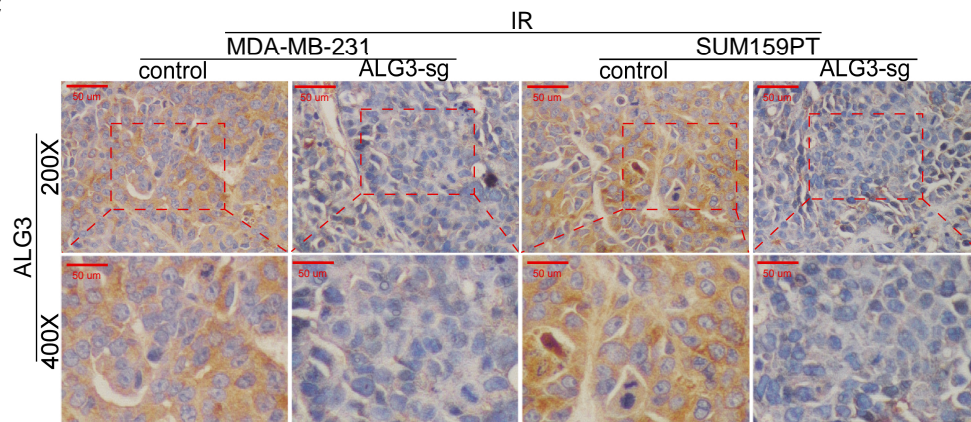**D**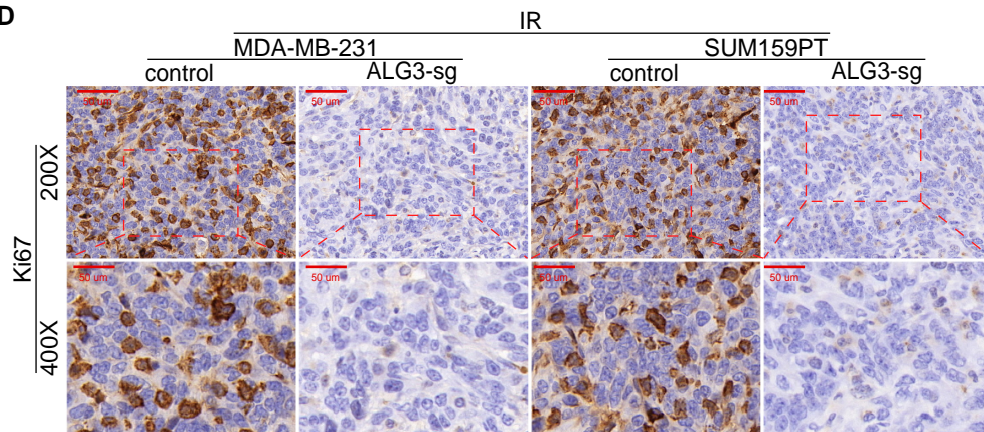**E**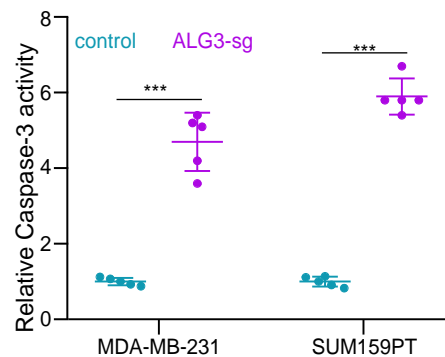

Supplement: Supplementary file 3 — Additional file 3: Supplementary Figure 3. (A) ALG3 expression after knocking out of ALG3 was confirmed in MDA-MB-231 and SUM159PT cell lines by Western blot. (B) Representative images of colony assays, with cells exposed to 0,2,4,6 Gy X ray treatment were as shown. (C) Immunohistochemistry image of tumor tissues from ALG3-sg mice depicts successful downregulation of ALG3. Original magnification, 200x and 400x. scale bar = 50 μm. (D) Immunohistochemistry images showed that Ki67 expression of ALG3-sg mice was downregulated. Original magnification, 200x and 400x. scale bar = 50 μm. (E) Cleaved caspase-3 activity was performed on tumor tissues from mice. *** P < 0.001. [file 13046_2021_1932_MOESM3_ESM.pdf]

**Fig. S4****A**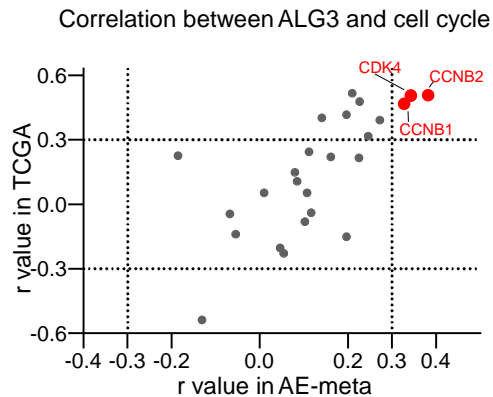**B**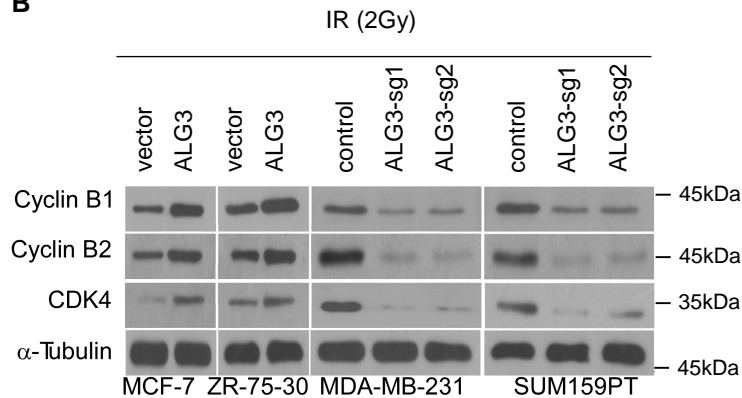**C**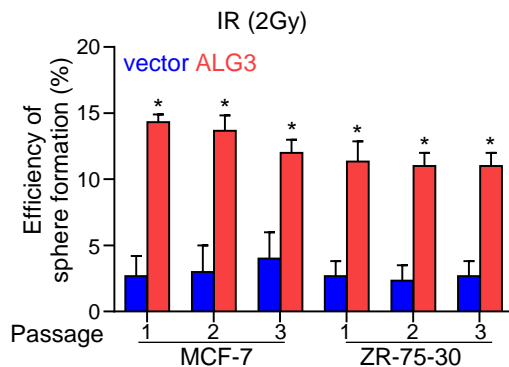**D**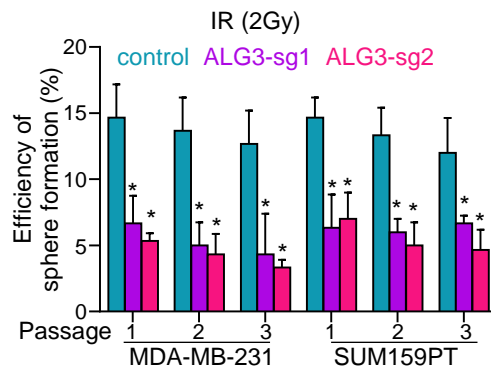

Supplement: Supplementary file 4 — Additional file 4: Supplementary Figure 4. (A) Analysis of correlation between ALG3 expression and cell cycle related genes based on TCGA database were performed. (B) Cyclin B1, cyclin B2, and CDK4 expression were up-regulated in ALG3-tansduced cells and down-regulated in ALG3-knocked out cells. α-Tubulin was detected as a loading control in Western blot. (C, D) Statistical graphs of secondary sphere formation assay in breast cancer cells were plotted. Each bar represents the mean ± SD of three independent experiments. All data were analyzed by Student’s t-test and one-way ANOVA with Tukey’s multiple comparison test. *P < 0.05. [file 13046_2021_1932_MOESM4_ESM.pdf]

**Fig. S5****A**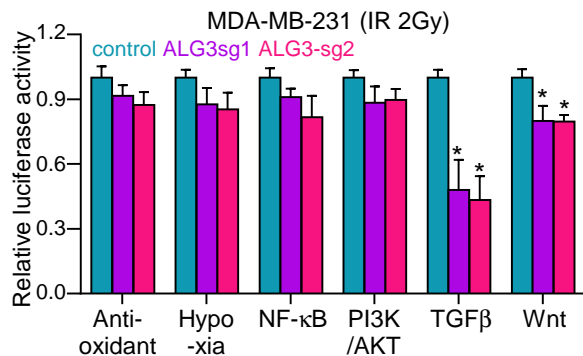**B**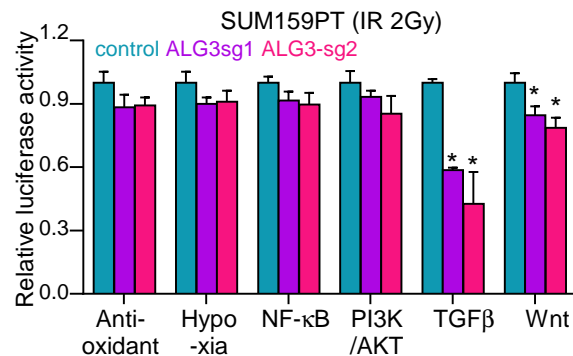**C**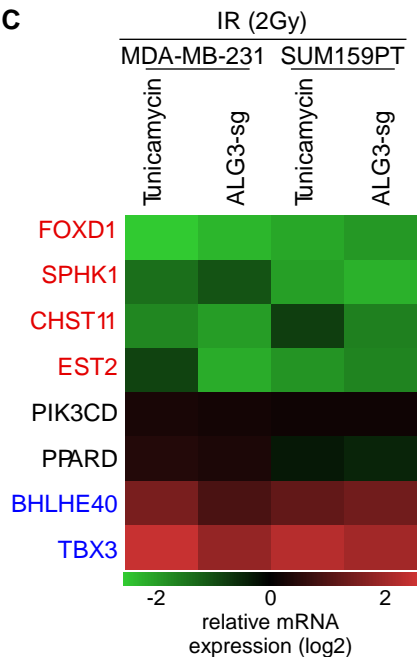**D**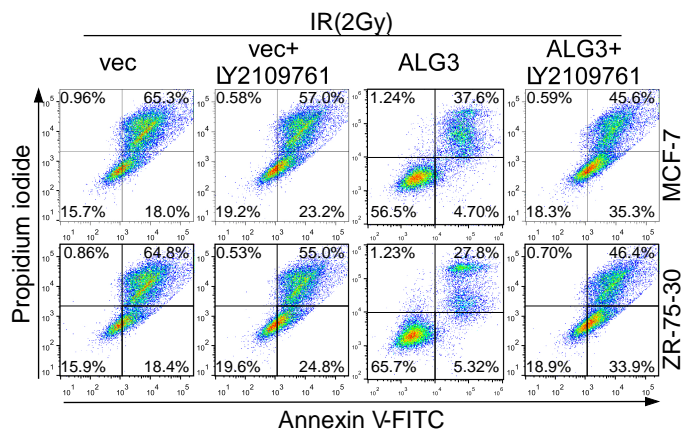**E**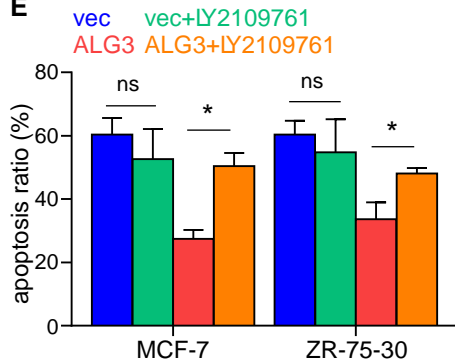**F**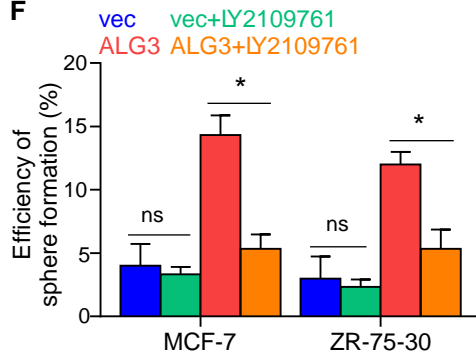**G**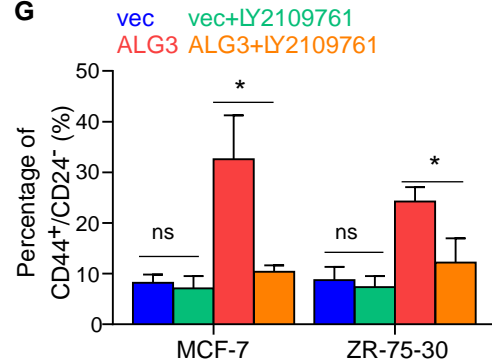

Supplement: Supplementary file 5 — Additional file 5: Supplementary Figure 5. (A, B) Radioresistance-associated pathways were examined by luciferase assay to find out the most influenced pathway after ALG3-knocked out. Each bar represents the mean ± SD of three independent experiments. All data were analyzed by one-way ANOVA with Tukey’s multiple comparison test. (C) mRNA expression level of several genes that regulated by TGF-β pathway in ALG3-sg cells or cells treated by tunicamycin compared with ALG3 control cells were detected by RT-PCR. GAPDH was detected as a loading control in RT-PCR. (D) Upregulation of ALG3 decreased the apoptosis ratio, whereas inhibition of TGFBR2 increased the apoptosis ratio of ALG3-transduced cells after radiation treatment, which were detected by flow cytometry. (E) Statistical graphs of apoptotic cells proportion in the vector, ALG3-vector +LY2109761(TGFBR2 inhibitor), ALG3-transduced, and ALG3-transduced +LY2109761 cells were plotted. Each bar represents the mean ± SD of three independent experiments. Data were analyzed by Student’s t-test. (F) Upregulation of ALG3 led to increasing the number of spheres, while inhibition of TGFBR2 decreased the number of spheres in ALG3-overexpressing cells, which were detected by secondary sphere assay. Each bar represents the mean ± SD of three independent experiments. Data were analyzed by Student’s t-test. (G) Upregulation of ALG3 led to increasing proportion of the CD44+CD24−subpopulation, whereas inhibition of TGFBR2 decreased proportion of the CD44+CD24− subpopulation in ALG3-transduced cells. Each bar represents the mean ± SD of three independent experiments. All data were analyzed by Student’s t-test. *P < 0.05. [file 13046_2021_1932_MOESM5_ESM.pdf]

**Figure S6**

**A**

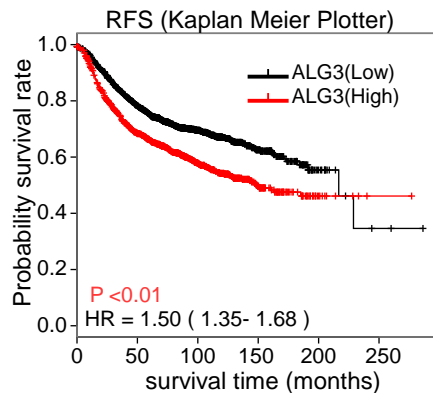

**B**

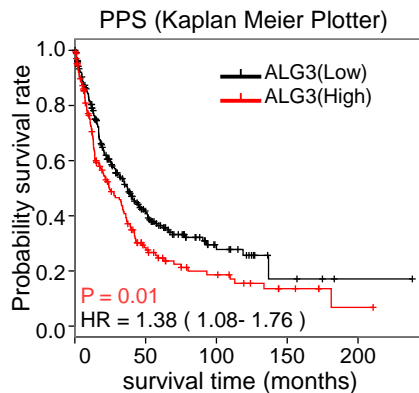

**C**

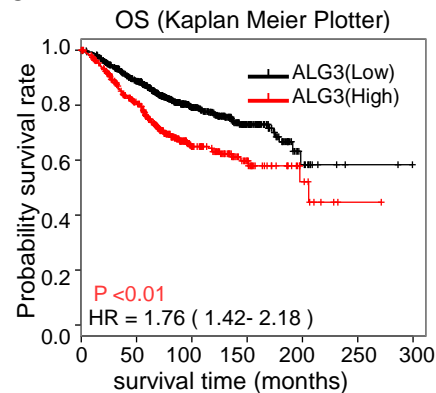

**D**

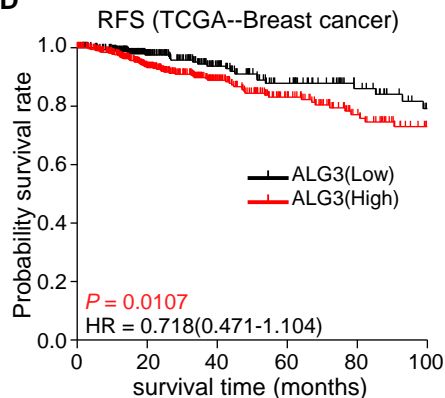

**E**

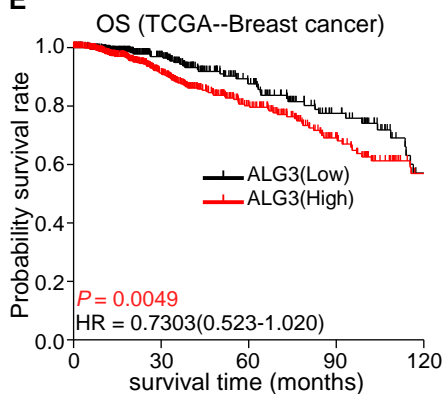

Supplement: Supplementary file 6 — Additional file 6: Supplementary Figure 6. (A–C) Kaplan–Meier analysis using the public breast cancer data sets showed that the ALG3 overexpression indicates shorter RFS, PPS, and OS. Website, https://kmplot.com/analysis/. (D, E) Kaplan–Meier analysis showed that ALG3 overexpression indicates shorter RFS and OS in the TCGA database. [file 13046_2021_1932_MOESM6_ESM.pdf]

**Fig. S7**

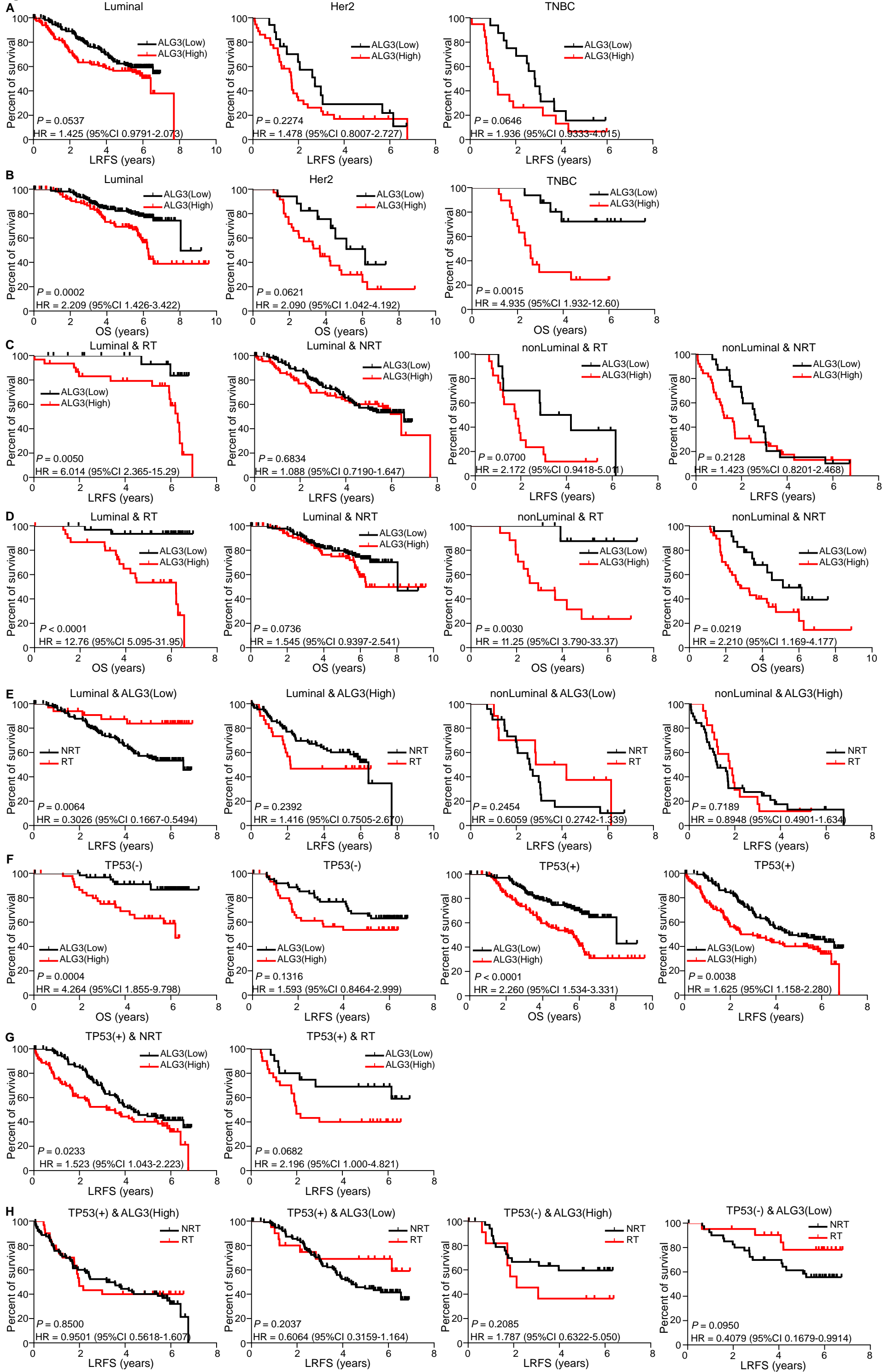

Supplement: Supplementary file 7 — Additional file 7: Supplementary Figure 7. (A-B) LRFS and OS of different tumor type patients, stratified by high and low ALG3 expression, were analyzed by Kaplan-Meier analysis with log-rank test. (C-D) Kaplan–Meier curves of LRFS and OS were plotted to evaluate the predictive value of ALG3 in patients grouped by tumor type and receiving radiotherapy or not. (E) Kaplan–Meier curves of LRFS were plotted to evaluate if radiotherapy contribute to prognosis of patients with ALG3 overexpressing in different tumor type. (F) LRFS and OS of patients with different p53 status, stratified by high and low ALG3 expression, were analyzed by Kaplan-Meier analysis with log-rank test. (G) LRFS of patients with mutational p53 who have received radiotherapy or not, stratified by ALG3 expression was analyzed by Kaplan-Meier analysis with log-rank test. (H) Kaplan–Meier curves of LRFS curves were plotted to evaluate if radiotherapy contribute to prognosis of patients with ALG3 overexpressing or with mutational p53. [file 13046_2021_1932_MOESM7_ESM.pdf]
