# Supplementary material for: ALG3 contributes to stemness and radioresistance through regulating glycosylation of TGF-β receptor II in breast cancer
Source: J Exp Clin Cancer Res. 2021 Apr 30;40:149. doi: 10.1186/s13046-021-01932-8 (PMC8086123; doi:10.1186/s13046-021-01932-8)
Supplement: Supplementary file 8 — Additional file 8: Table S1. Molecular baseline characteristics of patients. [file 13046_2021_1932_MOESM8_ESM.docx]

| **Patients** | **ER** | **PR** | **HER2** | **Ki67** | **p53 mutation** | **Tumor type** | **Tumor grade** |
| --- | --- | --- | --- | --- | --- | --- | --- |
| R-sen1 | - | + | - | + | + | Luminal | 2 |
| R-sen2 | - | + | +++ | - | - | Luminal | 3 |
| R-sen3 | ++ | + | - | - | + | Luminal | 2 |
| R-sen4 | +++ | + | - | - | - | Luminal | 1 |
| R-sen5 | + | ++ | - | - | - | Luminal | 2 |
| R-sen6 | ++ | ++ | - | - | - | Luminal | 2 |
| R-sen7 | ++ | ++ | +++ | - | - | Luminal | 2 |
| R-sen8 | + | - | - | - | - | Luminal | 2 |
| R-sen9 | +++ | +++ | - | ++ | - | Luminal | 2 |
| R-sen10 | +++ | +++ | ++ | - | - | Luminal | 2 |
| R-sen11 | + | +++ | + | ++ | - | Luminal | 2 |
| R-sen12 | - | - | - | - | + | Triple-negative | 2 |
| R-sen13 | + | - | ++ | - | - | Luminal | 1 |
| R-sen14 | +++ | - | - | - | - | Luminal | 2 |
| R-sen15 | + | + | - | - | - | Luminal | 2 |
| R-res16 | - | - | - | +++ | + | Triple-negative | 2 |
| R-res17 | - | - | - | - | + | Triple-negative | 3 |
| R-res18 | - | - | - | - | - | Triple-negative | 2 |
| R-res19 | + | + | - | - | + | Luminal | 2 |
| R-res20 | + | + | ++ | - | - | Luminal | 2 |
| R-res21 | - | - | ++ | ++ | + | HER2 | 2 |
| R-res22 | - | ++ | +++ | ++ | - | Luminal | 1 |
| R-res23 | - | + | - | ++ | + | Luminal | 2 |
| R-res24 | ++ | - | - | ++ | + | Luminal | 1 |
| R-res25 | + | ++ | - | - | - | Luminal | 2 |
| R-res26 | - | - | - | - | + | Triple-negative | 2 |
| R-res27 | + | + | +++ | - | - | Luminal | 2 |
| R-res28 | - | - | +++ | + | + | HER2 | 2 |
| R-res29 | + | + | - | ++ | - | Luminal | 2 |
| R-res30 | - | - | - | ++ | + | Triple-negative | 2 |

**Table S1 Molecular baseline characteristics of patients.**R-sen, sensitive to radiation; R-res, resistant to radiation.
