# Supplementary material for: ALG3 contributes to stemness and radioresistance through regulating glycosylation of TGF-β receptor II in breast cancer
Source: J Exp Clin Cancer Res. 2021 Apr 30;40:149. doi: 10.1186/s13046-021-01932-8 (PMC8086123; doi:10.1186/s13046-021-01932-8)
Supplement: Supplementary file 9 — Additional file 9: Table S2. Association of ALG3 expression with TP53 status and tumor type. [file 13046_2021_1932_MOESM9_ESM.docx]

| **Features of 30 samples** | **Radioresistant** | **Radiosensitive** | ***p* values** |
| --- | --- | --- | --- |
| **TP53 status** |  |  | 0.025 |
| Mutation | 9 | 3 |  |
| Wild type | 6 | 12 |  |
| **Tumor type** |  |  | 0.043 |
| Luminal | 8 | 14 |  |
| Her-2 | 2 | 0 |  |
| Triple-negative | 5 | 1 |  |

**Table S2** **Association of ALG3 expression with TP53 status and tumor type.**
