# Supplementary material for: ALG3 contributes to stemness and radioresistance through regulating glycosylation of TGF-β receptor II in breast cancer
Source: J Exp Clin Cancer Res. 2021 Apr 30;40:149. doi: 10.1186/s13046-021-01932-8 (PMC8086123; doi:10.1186/s13046-021-01932-8)
Supplement: Supplementary file 10 — Additional file 10: Table S3. The detail information of colony assay in MCF-7 cell line. [file 13046_2021_1932_MOESM10_ESM.docx]

**Table S3 The detail information of colony assay in MCF-7 cell line.**

| Radiation dose | SF (Mean ± SD^&^) SF (Mean ± SD^&^) | | *p*-values^*^ |
| --- | --- | --- | --- |
|  | Vector | ALG3 |  |
| 0 | 1.0000 ± 0.0000 | 1.0000 ± 0.0000 |  |
| 2 | 0.3623 ± 0.0495 | 0.7438 ± 0.0158 | 0.0029 |
| 4 | 0.0573 ± 0.0148 | 0.2547 ± 0.0324 | 0.0032 |
| 6 | 0.0068 ± 0.0020 | 0.0761 ± 0.0144 | 0.0128 |

^&^ Mean ± SD represents mean values of surviving fractions ± standard deviations

^*^*p* -values were calculated with a nonpaired Student's *t* test.
