# Supplementary material for: ALG3 contributes to stemness and radioresistance through regulating glycosylation of TGF-β receptor II in breast cancer
Source: J Exp Clin Cancer Res. 2021 Apr 30;40:149. doi: 10.1186/s13046-021-01932-8 (PMC8086123; doi:10.1186/s13046-021-01932-8)
Supplement: Supplementary file 11 — Additional file 11: Table S4. The detail information of colony assay in ZR-75-30 cell line. [file 13046_2021_1932_MOESM11_ESM.docx]

**Table S4 The detail information of colony assay in ZR-75-30 cell line.**

| Radiation dose | SF (Mean ± SD^&^) SF (Mean ± SD^&^) | | | *p*-values^*^ | |
| --- | --- | --- | --- | --- | --- |
|  | Vector | ALG3 | |  |  |
| 0 | 1.0000 ± 0.0000 | 1.0000 ± 0.0000 |  | |  |
| 2 | 0.2956 ± 0.0372 | 0.7349 ± 0.0329 |  | | 0.0001 |
| 4 | 0.0510 ± 0.0122 | 0.2403 ± 0.0237 |  | | 0.0002 |
| 6 | 0.0089 ± 0.0039 | 0.0750 ± 0.0267 |  | | 0.0132 |

^&^ & Mean ± SD represents mean values of surviving fractions ± standard deviations

^*^*p* -values were calculated with a nonpaired Student's *t* test.
