# Supplementary material for: ALG3 contributes to stemness and radioresistance through regulating glycosylation of TGF-β receptor II in breast cancer
Source: J Exp Clin Cancer Res. 2021 Apr 30;40:149. doi: 10.1186/s13046-021-01932-8 (PMC8086123; doi:10.1186/s13046-021-01932-8)
Supplement: Supplementary file 12 — Additional file 12: Table S5. The detail information of colony assay in MDA-MB-231 cell line. [file 13046_2021_1932_MOESM12_ESM.docx]

| Radiation dose | SF (Mean ± SD^&^) SF (Mean ± SD^&^) | | SF (Mean ± SD^&^) | *p*-values^*^(sg-1) | *p*-values^*^(sg-2) |
| --- | --- | --- | --- | --- | --- |
|  | Control | ALG3-sg1 | ALG3-sg2 |  |  |
| 0 | 1.0000 ± 0.0000 | 1.0000 ± 0.0000 | 1.0000 ± 0.0000 |  |  |
| 2 | 0.8050 ± 0.0776 | 0.3564 ± 0.0893 | 0.2826 ± 0.0665 | 0.0028 | 0.0009 |
| 4 | 0.3177 ± 0.0464 | 0.0773 ± 0.0357 | 0.0562 ± 0.0092 | 0.0021 | 0.0007 |
| 6 | 0.1073 ± 0.0138 | 0.0162 ± 0.0092 | 0.0107 ± 0.0013 | 0.0007 | 0.0003 |

**Table S5 The detail information of colony assay in MDA-MB-231 cell line.**

^&^ Mean ± SD represents mean values of surviving fractions ± standard deviations

^*^*p* -values were calculated with a nonpaired Student's *t* test.
