# Supplementary material for: ALG3 contributes to stemness and radioresistance through regulating glycosylation of TGF-β receptor II in breast cancer
Source: J Exp Clin Cancer Res. 2021 Apr 30;40:149. doi: 10.1186/s13046-021-01932-8 (PMC8086123; doi:10.1186/s13046-021-01932-8)
Supplement: Supplementary file 13 — Additional file 13: Table S6. The detail information of colony assay in SUM159PT cell line. [file 13046_2021_1932_MOESM13_ESM.docx]

**Table S6 The detail information of colony assay in SUM159PT cell line.**

| Radiation dose | SF (Mean ± SD^&^) SF (Mean ± SD^&^) | | SF (Mean ± SD^&^) | *p*-values^*^(sg-1) | *p*-values^*^(sg-2) |
| --- | --- | --- | --- | --- | --- |
|  | Control | ALG3-sg1 | ALG3-sg2 |  |  |
| 0 | 1.0000 ± 0.0000 | 1.0000 ± 0.0000 | 1.0000 ± 0.0000 |  |  |
| 2 | 0.7131 ± 0.0310 | 0.3000 ± 0.0620 | 0.3625 ± 0.0350 | 0.0005 | 0.0002 |
| 4 | 0.2545 ± 0.0105 | 0.0646 ± 0.0165 | 0.0745 ± 0.0176 | 0.0001 | 0.0001 |
| 6 | 0.0811 ± 0.0108 | 0.0128 ± 0.0040 | 0.0107 ± 0.0046 | 0.0005 | 0.0005 |

^&^ Mean ± SD represents mean values of surviving fractions ± standard deviations

^*^*p* -values were calculated with a nonpaired Student's *t* test.
