# Supplementary material for: ALG3 contributes to stemness and radioresistance through regulating glycosylation of TGF-β receptor II in breast cancer
Source: J Exp Clin Cancer Res. 2021 Apr 30;40:149. doi: 10.1186/s13046-021-01932-8 (PMC8086123; doi:10.1186/s13046-021-01932-8)
Supplement: Supplementary file 14 — Additional file 14: Table S7. The number of patients in subgroups. [file 13046_2021_1932_MOESM14_ESM.docx]

| **Subgroups** | **RT^*^** | **NRT^&^** | **ALG3(high)** | **ALG3(low)** |
| --- | --- | --- | --- | --- |
| **Luminal subtype** | 64 | 224 | 118 | 170 |
| **Non-Luminal** | 27 | 61 | 55 | 33 |
| **p53 positive** | 50 | 202 | 117 | 135 |
| **p53 negative** | 33 | 76 | 47 | 62 |

**Table S7 The number of patients in subgroups.**

^*^RT represents radiotherapy; ^&^NRT represents not received radiotherapy.
